# Scutellarin regulates microglia-mediated TNC1 astrocytic reaction and astrogliosis in cerebral ischemia in the adult rats

**DOI:** 10.1186/s12868-015-0219-6

**Published:** 2015-11-25

**Authors:** Ming Fang, Yun Yuan, Parakalan Rangarajan, Jia Lu, Yajun Wu, Huadong Wang, Chunyun Wu, Eng-Ang Ling

**Affiliations:** Department of Emergency and Critical Care, Guangdong General Hospital, Guangdong Academy of Medical Sciences, Guangzhou, 510080 China; Department of Anatomy and Histology/Embryology, Faculty of Basic Medical Sciences, Kunming Medical University, 1168 West Chunrong Road, Kunming, 650500 Peoples’ Republic of China; Department of Anatomy, Yong Loo Lin School of Medicine, National University of Singapore, 4 Medical Drive, MD10, Singapore, 117594 Singapore; Defence Medical and Environmental Research Institute, DSO National Laboratories, 27 Medical Drive, Singapore, 117510 Singapore; Department of Pathophysiology, School of Medicine, Jinan University, Guangzhou, 510632 China

**Keywords:** Scutellarin, Cerebral ischemia, Astrogliosis, GFAP, Notch, Nestin, Proinflammatory mediators, TNC1

## Abstract

**Background:**

Scutellarin, an anti-inflammatory agent, effectively suppressed microglia activation in rats with middle cerebral artery occlusion (MCAO). Robust microglia activation, acute in onset, was followed by astrogliosis. This study was aimed to determine if scutellarin would also affect the reactive astrocytes that play an important role in tissue repair. Expression of GFAP and Notch-1 and its members: Notch receptor intracellular domain (NICD), and transcription factor hairy and enhancer of split-1 (HES-1), together with nestin and proinflammatory mediators was assessed by immunofluorescence staining in TNC1 astrocytes treated, respectively, with BV-2 conditioned medium (CM) and CM + lipopolysaccharide (LPS) (CM + L) serving as the controls, and conditioned medium derived from LPS-activated BV-2 cells pretreated with scutellarin (CM + SL). Study of the above biomarkers was then extended to reactive astrocytes in scutellarin injected MCAO rats.

**Results:**

TNC1 astrocytes remained relatively unreactive in terms of expression of different biomarkers to direct scutellarin treatment when compared with the control cells. In comparison to cells in the control medium (CM, CM + L), they responded vigorously to CM + SL as evidenced by the enhanced protein expression of GFAP, Notch-1, NICD and HES-1 coupled with that of nestin, TNF-α, IL-1β, and iNOS by Western and immunofluorescence analysis. Electron microscopy showed marked hypertrophy and cell expansion of TNC1 astrocytes bearing many filamentous processes indicative of enhanced astrocyte reaction when treated with CM + SL. In MCAO rats, scutellarin also augmented the expression of the above markers in reactive astrocytes; moreover, astrocytes were evidently hypertrophic.

**Conclusions:**

The results suggest that scutellarin regulates astrogliosis; more importantly, it is microglia-mediated as demonstrated in vitro. Increased expression of Notch signaling in synchrony with nestin may be linked to proliferation and “de-differentiation” of reactive astrocytes; the significance of enhanced TNF-α, IL-1β and iNOS expression in reactive astrocytes by scutellarin may be neuroprotective but this remains speculative.

**Electronic supplementary material:**

The online version of this article (doi:10.1186/s12868-015-0219-6) contains supplementary material, which is available to authorized users.

## Background

Scutellarin (4,5,6-trihydroxyflavone-7-glucuronide) is the major active component extracted from *Erigeron breviscapus*. It is a Chinese herbal compound endowed with antioxidant and anti-inflammation properties [1–4]. In the brain, scutellarin has been shown to decrease microglia inflammatory response [4]. In addition to its antioxidant and anti-inflammatory properties, scutellarin has been demonstrated to have anti-apoptotic properties in animal models of ischemic stroke [3]. We reported recently that scutellarin effectively suppressed production of proinflammatory mediators in activated microglia in experimentally induced cerebral ischemia following middle cerebral artery occlusion (MCAO) in the adult rats and in BV-2 microglia in vitro [5, 6]. It was shown that scutellarin decreased the production of proinflammatory cytokines including TNF-α and IL-1β and reactive oxygen species (ROS) by activated microglia whose robust reaction featured prominently in the acute phase of cerebral ischemia. All this has pointed to the therapeutic potential of scutellarin and its clinical use for amelioration of microglia-mediated neuroinflammation.

While activated microglia evidently preponderated in the ischemic cerebral tissue in the early phase of MCAO [5, 6], massive astrocytes soon accumulated the infarcted areas. Indeed cerebral ischemia leads to widespread progressive alterations in astrocytes, including cell hypertrophy, upregulation of intermediate filaments and increase in cell proliferation, commonly referred to as reactive astrogliosis [7–11]. The close spatial relation between activated microglia and reactive astrocytes in the ischemic area invites speculation that both glial cells might work in concert through cellular interaction or communication in the ensuing healing process. The functional relationship between activated microglia and reactive astrocytes, however, has remained to be fully explored. In consideration of scutellarin as a potential therapeutic agent, it is therefore desirable to determine if it would also act on astrocytes as for activated microglia, and if so, the mode of its action on astrocytes.

This study was therefore aimed to determine whether scutellarin would exert its effects on astrocytes that are closely associated with activated microglia. In the latter, we have reported that expression of pro-inflammatory mediators and Notch-1 pathway and its members was significantly suppressed by scutellarin. It is relevant therefore to ascertain if these biomarkers strongly expressed in reactive astrocytes would also be affected. Secondly, if indeed scutellarin were able to affect or regulate the astrocyte reaction, a compelling question would be whether it would directly act on astrocytes, or indirectly such as via activated microglia. In the latter, the notion took into consideration of the reactive response of microglia and astrocytes in temporal sequence [12, 13] in cerebral ischemia, in which acute microglial activation appears to precede that of astrocyte reaction; furthermore, both glial cells are spatially related. We report here that scutellarin could enhance the expression of GFAP, nestin, Notch-1 signaling and its members in TNC1 astrocytes as well as in reactive astrocytes in MCAO rats. More strikingly, scutellarin was found to amplify the expression of above-named biomarkers along with proinflammatory mediators, a process that is mediated by activated microglia as demonstrated in vitro.

## Results

### TNC1 viability assay with scutellarin

**Fig. 1:**
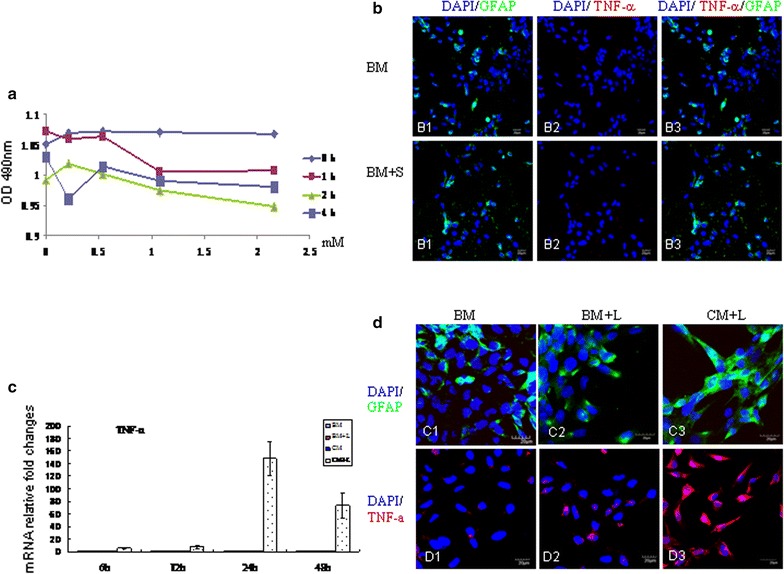
Cell viability assay of TNC1 astrocytes (**a**): scutellarin (in the range of 0.2–2.0 mM) incubated for different duration did not result in any significant cell death. Treatment of TNC1 with scutellarin (**b**): scutellarin at 0.54 mM did not elicit a noticeable reaction in TNC1 whose GFAP/TNF-α (*B1*–*3*) expression remained comparable to cells in the control in basic medium (BM) (*A1*–*3*). BM + S, basic medium + scutellarin. Microglia mediate TNC1 astrocyte reaction (**c**): TNF-α mRNA expression in TNC1 astrocytes remained relatively unchanged at all time-points following treatment with BM, BM + L and BV-2 conditioned medium (CM). However, in TNC1 incubated CM + L for various time points, TNC1 showed a remarkable increase in TNF-α peaking at 24 h. Confocal images in **d** showing GFAP (*C1*–*3*), and TNF-α (*D1*–*3*) expression in TNC1 astrocytes incubated with different medium for 24 h. Compared with cells incubated in BM and BM + LPS (BM + L), TNC1 astrocytes incubated with LPS-stimulated BV-2 cell conditioned medium (CM + L) (*C3*, *D3*) are hypertrophic and exhibit a marked increase in GFAP and TNF-α immunofluorescence. *Scale bars* 20 μm

TNC1 astrocyte cell line instead of primary cultured astrocytes was used for this study to ensure that we obtained enough cells for adequate amounts of protein for Western blot analysis. The cytotoxicity data was obtained by the MTS assay for the effect of scutellarin on TNC1 cells. Scutellarin (in the range of 0.2–2.0 mM) did not result in any significant cell death (Fig. 1a). In this study, we have used scutellarin at 0.54 mM for all subsequent analysis. This dosage was used for treatment of BV-2 cells also in our previous studies in which dose dependent assay had earlier been done [5, 6].

### Scutellarin does not exert a direct effect on TNC1

To determine if scutellarin would exert a direct effect on TNC1, it was added directly into the medium with TNC1. By phase contrast microscopy, the external morphology of TNC1 astrocytes incubated in the basic medium + 0.54 mM scutellarin (BM + S) appeared comparable to that in the control cells in the basic medium (BM). Furthermore, TNF-α (Fig. 1b: compared B1–3 with A1–3) and iNOS (Additional file 1a) immunofluorescence in BM + S remained relatively unaltered as compared with the control cells incubated in BM. This indicates that scutellarin does not stimulate TNC1 directly.

### Lipopolysaccharide (LPS) does not exert a direct effect on TNC1

By RT-PCR, TNF-α (Fig. 1c) and iNOS (Additional file 1b) mRNA expression levels in TNC1 astrocytes incubated in the basic medium (BM), BM + LPS (BM + L) and BV-2 conditioned medium (CM) were negligible. Remarkably, the mRNA expression levels were drastically increased by several hundred folds when treated with LPS-stimulated BV-2 conditioned medium (CM + L) (Fig. 1c; Additional file 1b). This shows that TNC1 activation was only mildly elicited and directly by LPS; instead, its vigorous activation is mediated by activated microglia. TNF-α, IL-1β and iNOS were used as markers for reactive astrocytes along with GFAP because these proinflammatory mediators have been reported to be highly expressed in activated astrocytes [14, 15].

### TNC1 activation is mediated by activated microglia

Microglia-mediated activation of TNC1 astrocytes was further evidenced by the drastic increase in GFAP (Fig. 1d: C1–C3), TNF-α (Fig. 1d: D1–D3) and iNOS (Additional file 1c: C1–C3) immunofluorescence in cells incubated in CM + L compared with cells incubated in BM and BM + L groups. Furthermore, TNC1 cells treated with CM + L appeared evidently hypertrophic (Fig. 1d: C3, D3).

### Scutellarin upregulates GFAP, Notch-1, NICD, HES and nestin expression in TNC1 via BV-2-conditioned medium

**Fig. 2:**
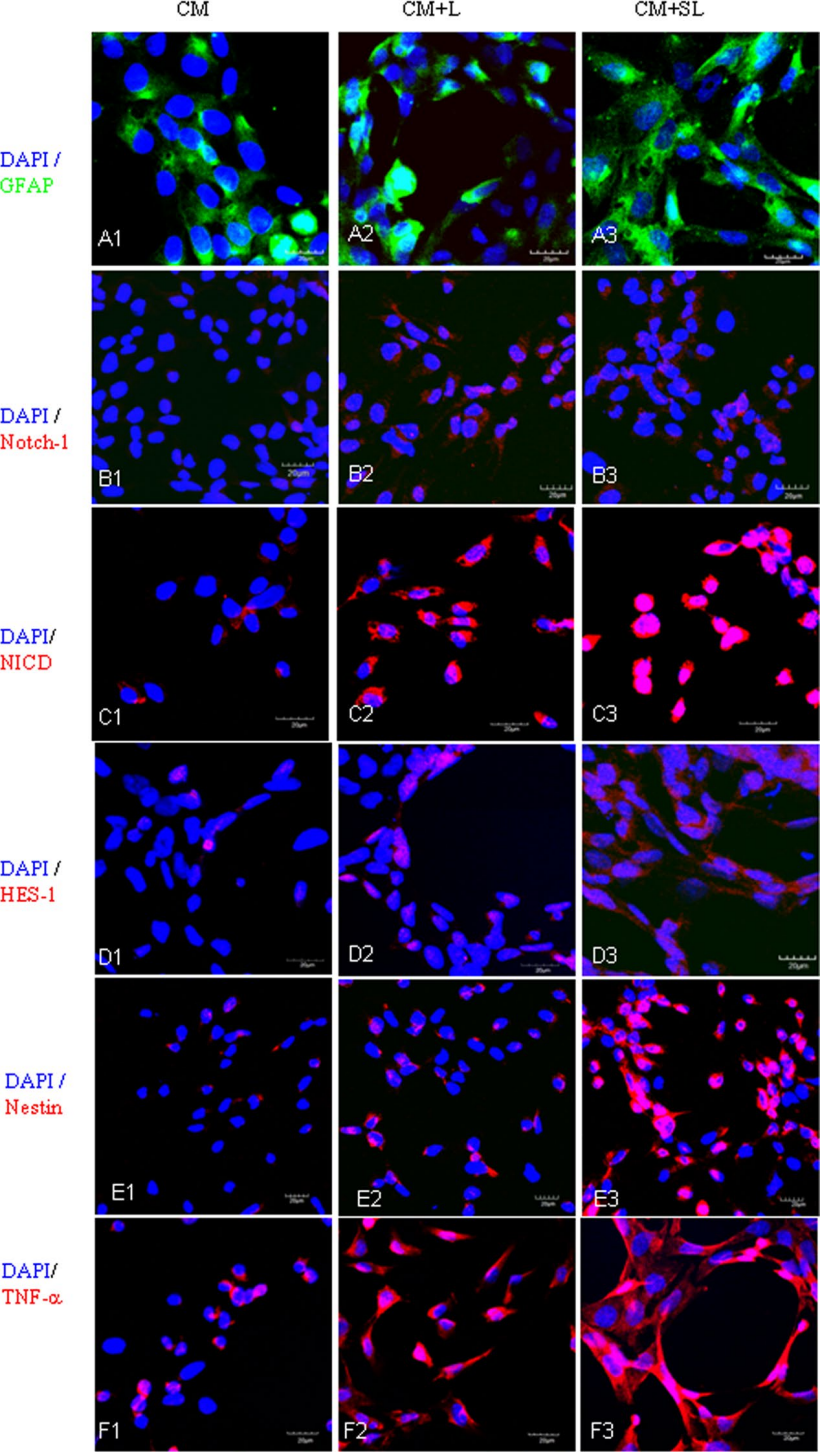
Scutellarin enhanced GFAP (**A1**–**A3**), Notch-1 (**B1**–**B3**), NICD (**C1**–**C3**), HES-1 (**D1**–**D3**), nestin (**E1**–**E3**) and TNF-α (**A1**–**3**) expression in TNC1 via BV-2-conditioned medium. Moderate GFAP expression was detected in TNC1 incubated in CM; also, Notch-1, NICD, HES-1, nestin and TNF-α (**B1**, **C1**, **D1**, **E1**, **F1**) was weakly expressed. The expression however was enhanced in CM + L (**B2**, **C2**, **D2**, **E2**, **F2**). Upon treatment with CM + SL for 24 h, expression of all markers was drastically increased being more pronounced for NICD (**C3**) and nestin (**E3**). Note TNC1 astrocytes project long cytoplasmic processes with expansions (**A3**, **D3**, **F3**). *Scale bars* 20 μm

GFAP was moderately expressed in TNC1 incubated in CM (a1, Fig. 2); it was enhanced in TNC1 treated with CM + L (a2, Fig. 2). Notch-1, NICD, HES and nestin expression was hardly detected in CM group (Fig. 2B1, C1, D1, E1), but was enhanced in CM + L (b2, c2, d2, e2). Remarkably, upon pretreatment with scutellarin in CM + SL for 24 h, expression of all markers was drastically increased being more pronounced for NICD (Fig. 2C3) and nestin (Fig. 2E3).

### Scutellarin upregulates TNF-α, IL-1β and iNOS expression in TNC1 via BV-2-conditioned medium

In TNC1 incubated in CM, the cells exhibited moderate TNF-α expression (Fig. 2F1) which was augmented in CM + L (F2). When treated with CM + SL, the expression was further enhanced (Fig. 2F3). A similar pattern in expression changes was observed for IL-1β and iNOS (Additional file 2). A striking morphological change in TNC1 astrocytes treated with CM + SL was the extension of long cytoplasmic processes emanated from the hypertrophic cell body (Fig. 2F3; Additional file 2B3). By contrast, the cells in CM appeared more rounded, and those in CM + L showed only a few short processes.

### Western blot analysis of GFAP, Notch-1, NICD, HES-1, TNF-α, IL-1β and iNOS in TNC1 in different treatments

**Fig. 3:**
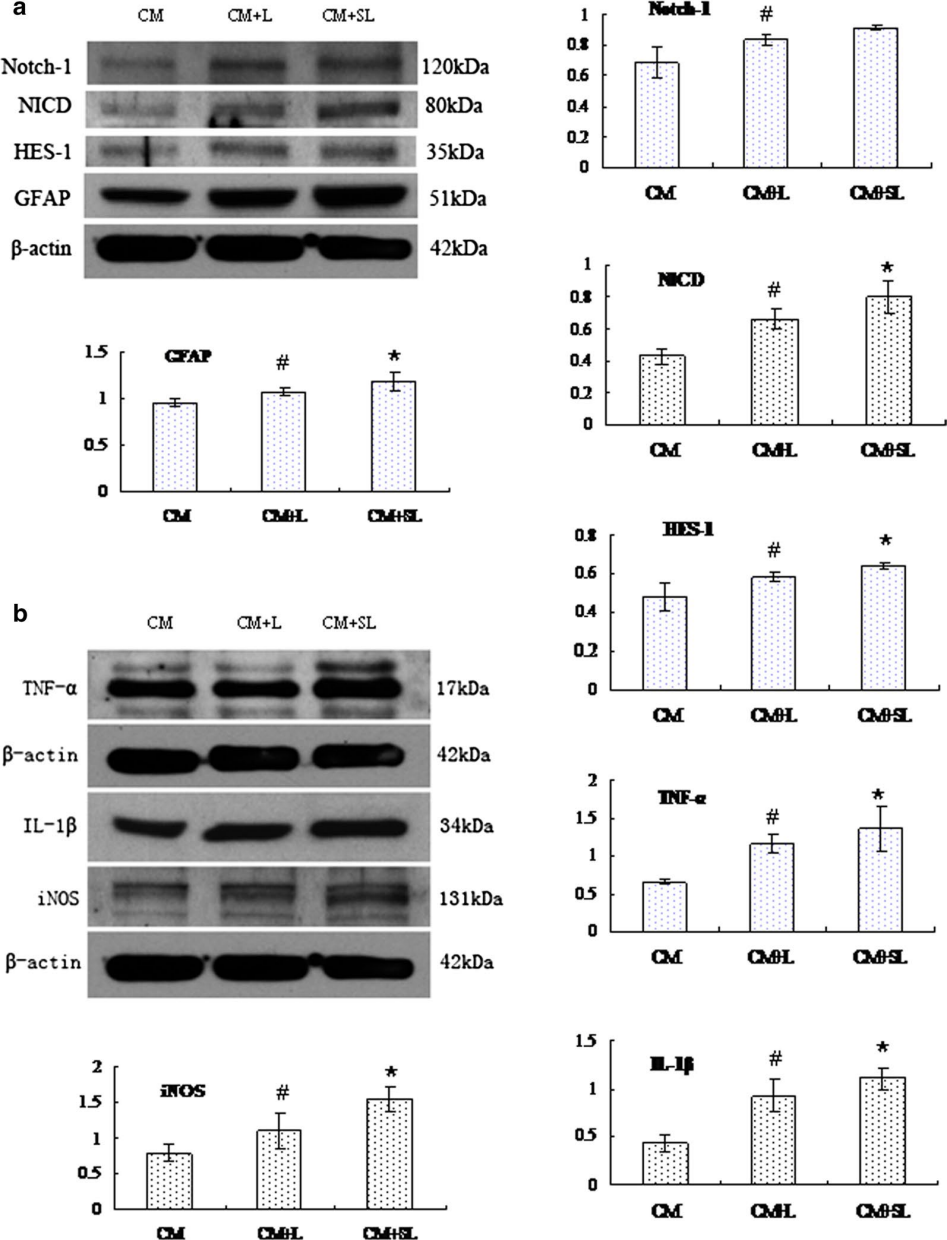
Protein expression of GFAP, Notch-1, NICD, HES-1, TNF-α, IL-1β and iNOS in TNC1 following different treatments. **a** Expression levels of GFAP, Notch-1, NICD, and HES-1 were significantly increased after treatment with CM + L when compared with the control in CM; likewise, the expression of TNF-α, IL-1β and iNOS was significantly increased. **b** In CM + SL treated TNC1, the expression levels of the above markers were further elevated as compared with that of CM + L treated cells. *Bar graphs* represent expression changes of the respective markers. Significant differences in protein levels are expressed as *^#^P < 0.05. The values represent the mean ± SD in triplicate.^ #^CM + L vs CM, *CM + SL vs CM + L

The expression level of GFAP (51 kDa), Notch-1 (120 kDa), NICD (80 kDa), and HES-1 (35 kDa) was significantly increased after treatment with CM + L when compared with the control in CM (Fig. 3a); likewise, the expression of TNF-α, IL-1β and iNOS was significantly increased (Fig. 3b). In CM + SL treated TNC1, the expression levels of the above proteins were further elevated as compared with that of CM + L treated cells. Expression changes of the respective proteins are indicated against the corresponding bar graphs (Fig. 3). The optical density values (mean ± SD) of each marker are given in Table 1.

**Table 1:** Optical density values of each protein marker (mean ± SD)

	Notch-1	NICD	HES-1	TNF-α	IL-1β	iNOS	GFAP
CM	0.68 ± 0.10	0.42 ± 0.04	0.48 ± 0.07	0.65 ± 0.03	0.43 ± 0.08	0.78 ± 0.12	0.96 ± 0.04
CM + L	0.83 ± 0.03^#^	0.66 ± 0.06^#^	0.58 ± 0.02^#^	1.16 ± 0.11^#^	0.93 ± 0.16^#^	1.09 ± 0.26^#^	1.07 ± 0.04^#^
CM + SL	0.92 ± 0.02	0.80 ± 0.09*	0.65 ± 0.01*	1.36 ± 0.19*	1.13 ± 0.11*	1.55 ± 0.16*	1.18 ± 0.09*

#* *P* < 0.05; ^#^ CM + L vs CM, * CM + SL vs CM + L

### Electron microscopy

**Fig. 4:**
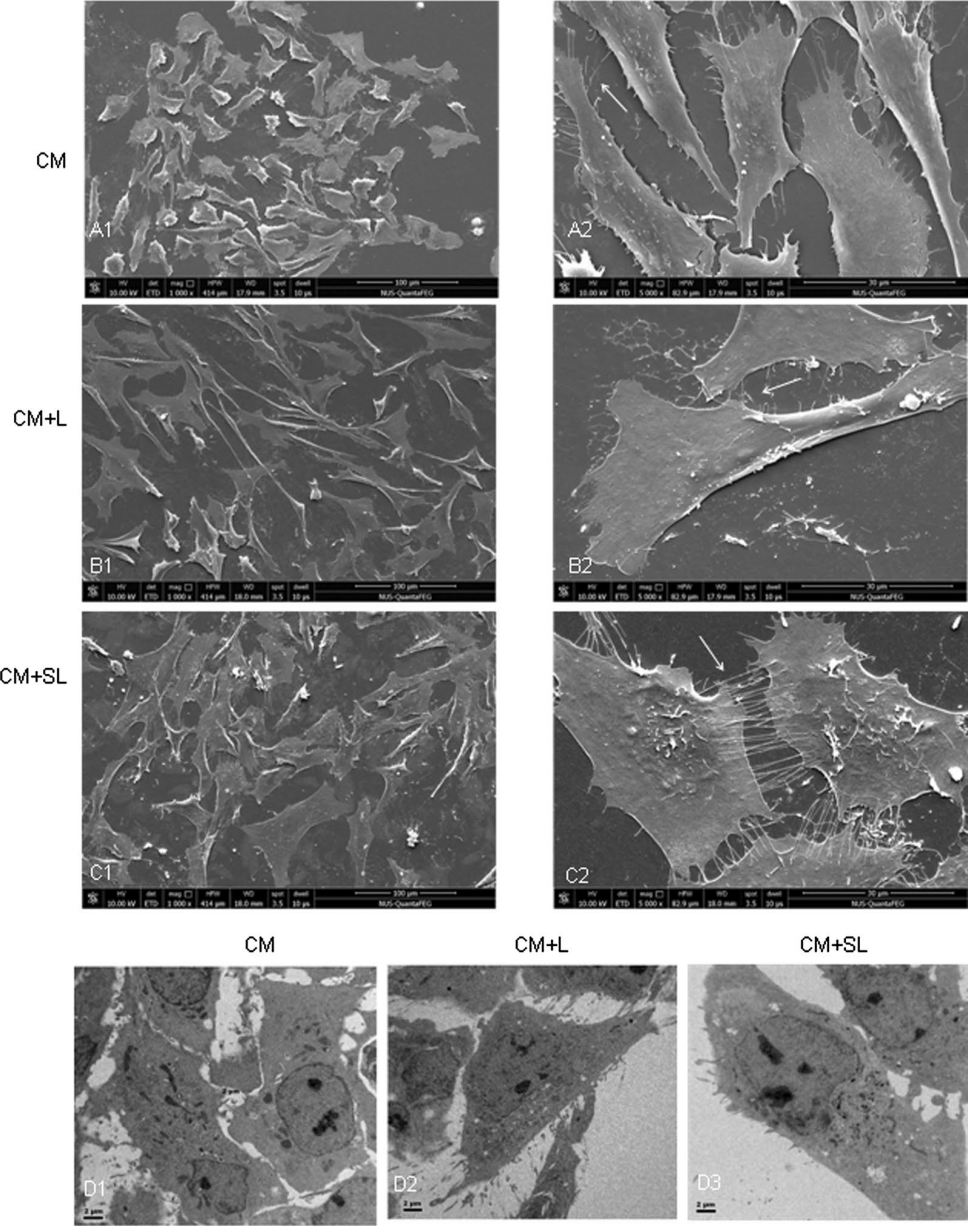
Scanning electron microscopy of TNC1 astrocytes in CM (**A1–2**), CM + SL (**B1–2**) and CM + SL (**C1–2**) groups. Note the drastic transformation of TNC1 from oblong outline (CM, **A1–2**) to “squamous” appearance in the CM + SL (**C1–2**) whose cell surface exhibit a large number of filamentous processes (*arrows*). By transmission electron microscopy (**D1–3**), TNC1 astrocytes in CM + SL are evidently enlarged (**D3**) as compared with CM (**D1**) and CM + L (**D2**) groups; moreover, the cells contain a larger amount of cytoplasm rich in polyribosomes and usual organelles

By scanning electronic microscopy, most TNC1 astrocytes in CM group were oblong; on closer examination, the surface projected some filamentous processes (Fig. 4a). In CM + L group, the cells became hypertrophic bearing long extending processes (Fig. 4b) when compared with cells in the CM group; the processes in some cells were expanded (Fig. 4b). Most of the cells in CM + SL group were grossly hypertrophic (Fig. 4c); large numbers of filamentous processes projected from the cell surface and appeared to intertwine with those emanated from the adjacent cells (Fig. 4c). Most strikingly, TNC1 astrocytes in CM + SL spread out extensively and thinly giving them a “squamous” appearance. In transmission electron microscopy, TNC1 astrocytes in all three groups showed a round nucleus bearing one to three conspicuous nucleoli (Fig. 4d). The copious cytoplasm in CM + SL group as compared with the other two groups was filled with polyribosomes and the usual cell organelles (Fig. 4d). Compared with other groups, cells in CM + SL had a larger cell profile. Many long filamentous processes projected from the cell surface as observed by scanning electron microscopy.

### Morphological evidence of interaction between activated microglia and reactive astrocytes

**Fig. 5:**
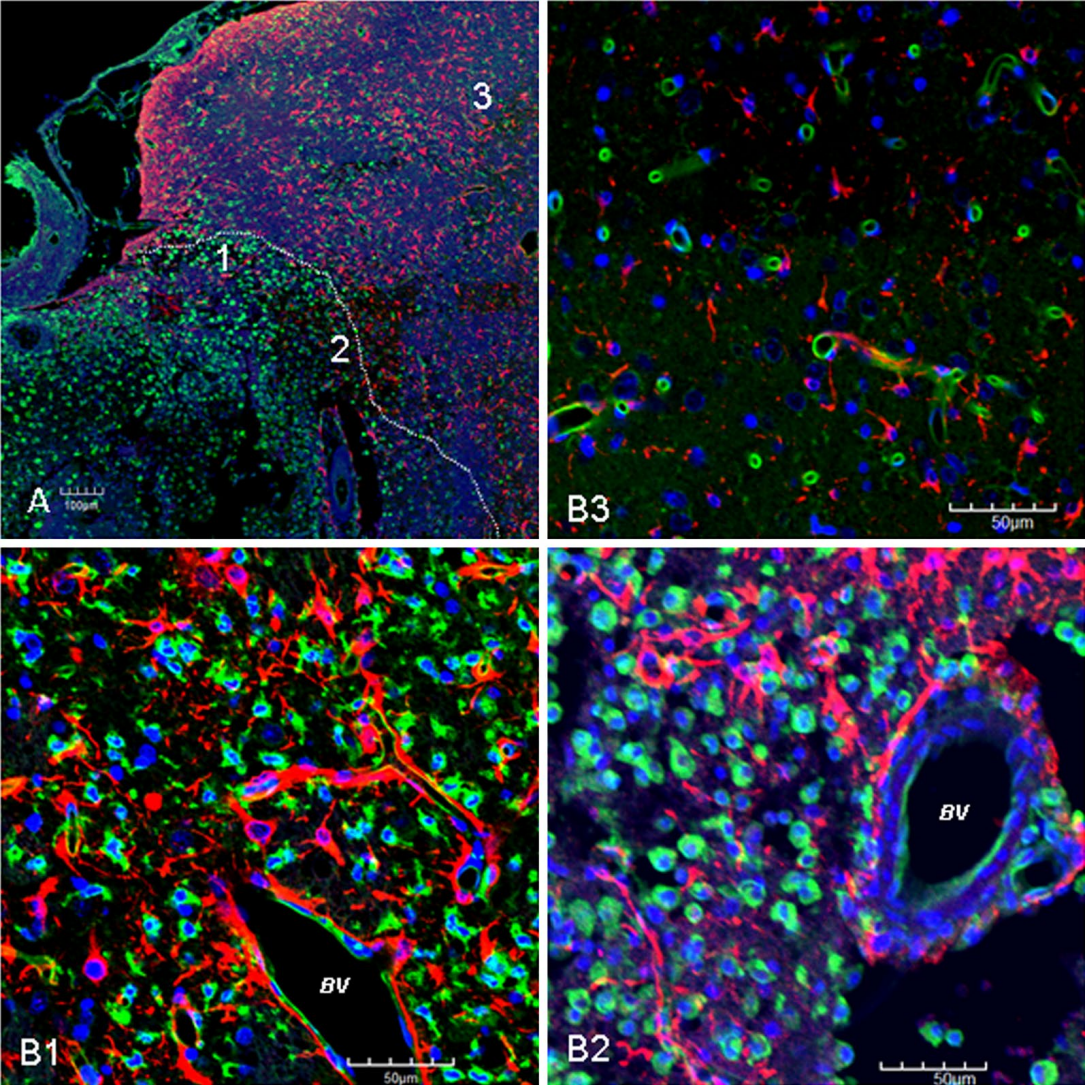
Morphological evidence of interaction between activated microglia and reactive astrocytes. Figure showing ipsilateral ischemic cerebral cortex at 3 days after MCAO. The *dotted line* in **A** delineates approximately the border of the infarct epicenter which shows massive accumulation of activated microglia intermingled with reactive astrocytes. **B1**, **B2** show lectin-labeled activated microglia (*green*). They are obviously hypertrophic and are mostly rounded (**B1**) or emit short stout processes (**B2**). GFAP labeled reactive astrocytes (*red*) are also hypertrophic, and extend their long processes between the activated microglia; in some areas, they are closely associated (**B1**, **B2**). Astrocytic processes also surround the blood vessel (BV) in **B1**, **B2**. The close spatial relation between the activated microglia and reactive astrocytes is not evident away from the border (**B3**). **B1**–**3** are enlarged view of area *1–3* in **A**. *Scale bar* in **A**: 100 μm; *scale bar* in **B**: 50 μm. DAPI, *blue*

In rats subjected to MCAO, a large infarcted area was observed in the ipsilateral cerebrum at early time-points. By immunofluorescence labeling, the epicenter of the infarcted area at 3 and 7 days after MCAO was occupied by massive activated microglia labeled by lectin (Fig. 5). On closer examination, the lectin labeled activated microglia were mostly round with some cells emitted short cytoplasmic processes (Fig. 5). At the border area with the non-lesion cortex or adjacent to it (penumbral area), activated microglia were admixed with GFAP positive astrocytes that appeared hypertrophic with long projecting and broad processes (Fig. 5); in some areas, both glial cell types were closely associated or intermingled. In non-lesion area, microglia and astrocytes appeared relatively normal both bearing fine processes; furthermore, their close spatial relation as observed in the lesion zone was not evident (Fig. 5).

### Scutellarin enhanced astrocyte reaction in ischemic cortex

**Fig. 6:**
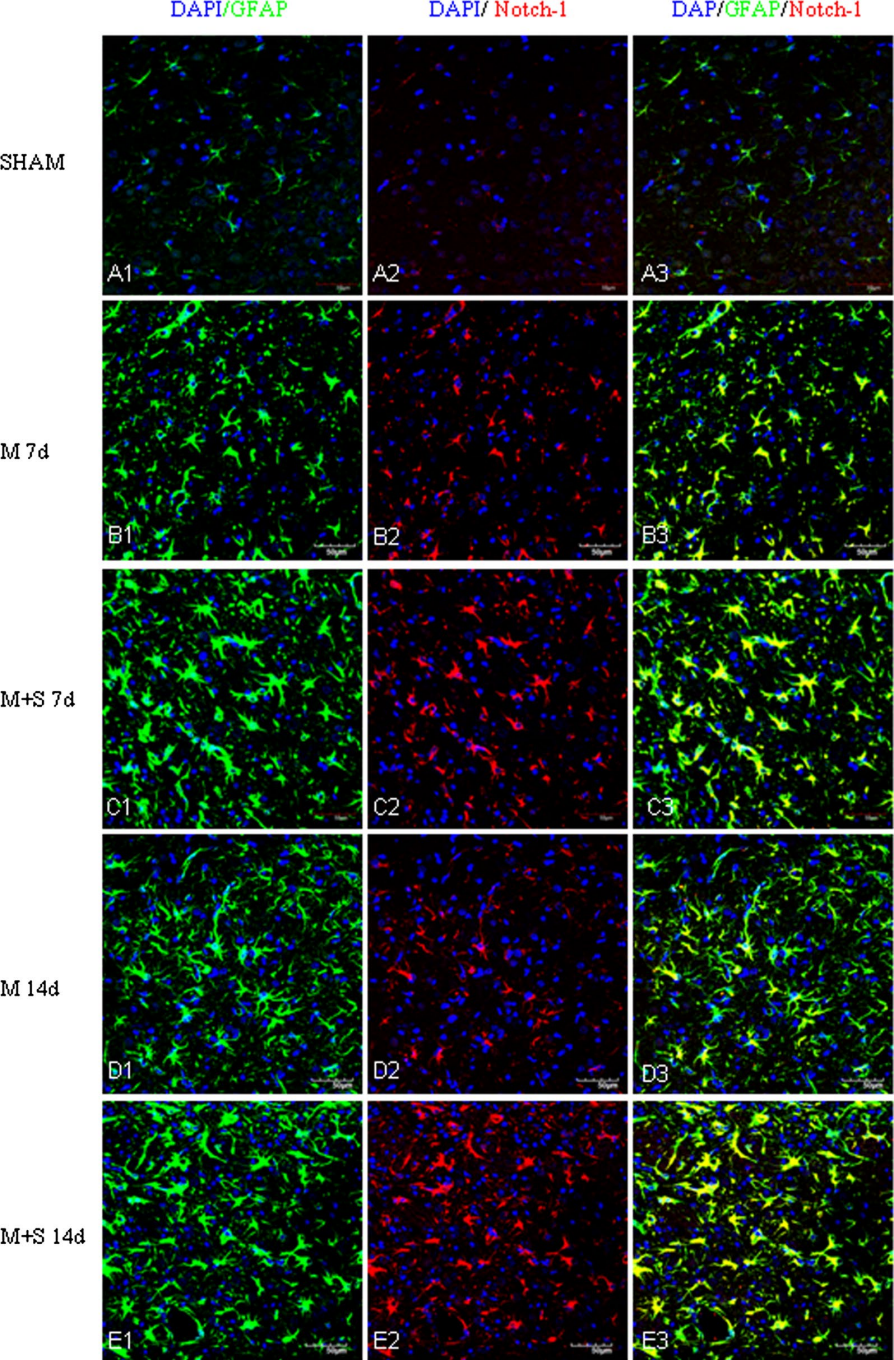
Scutellarin enhanced Notch-1expression in astrocytes after MCAO. Notch-1 expression is negligible in astrocytes in the sham (**A1**–**3**). Its expression (*red*) was moderately induced by MCAO in GFAP positive astrocytes (*green*) at 7 (**B1**–**3**) and 14 days (**D1**–**3**). However, Notch-1 expression was markedly increased in hypertrophic astrocytes in scutellarin treated MCAO rats (**C1**–**3**, **E1**–**3**) in comparison with the matching controls (**B1**–**3**, **D1**–**3**). *Scale bars*: 50 µm. DAPI-*blue*

**Fig. 7:**
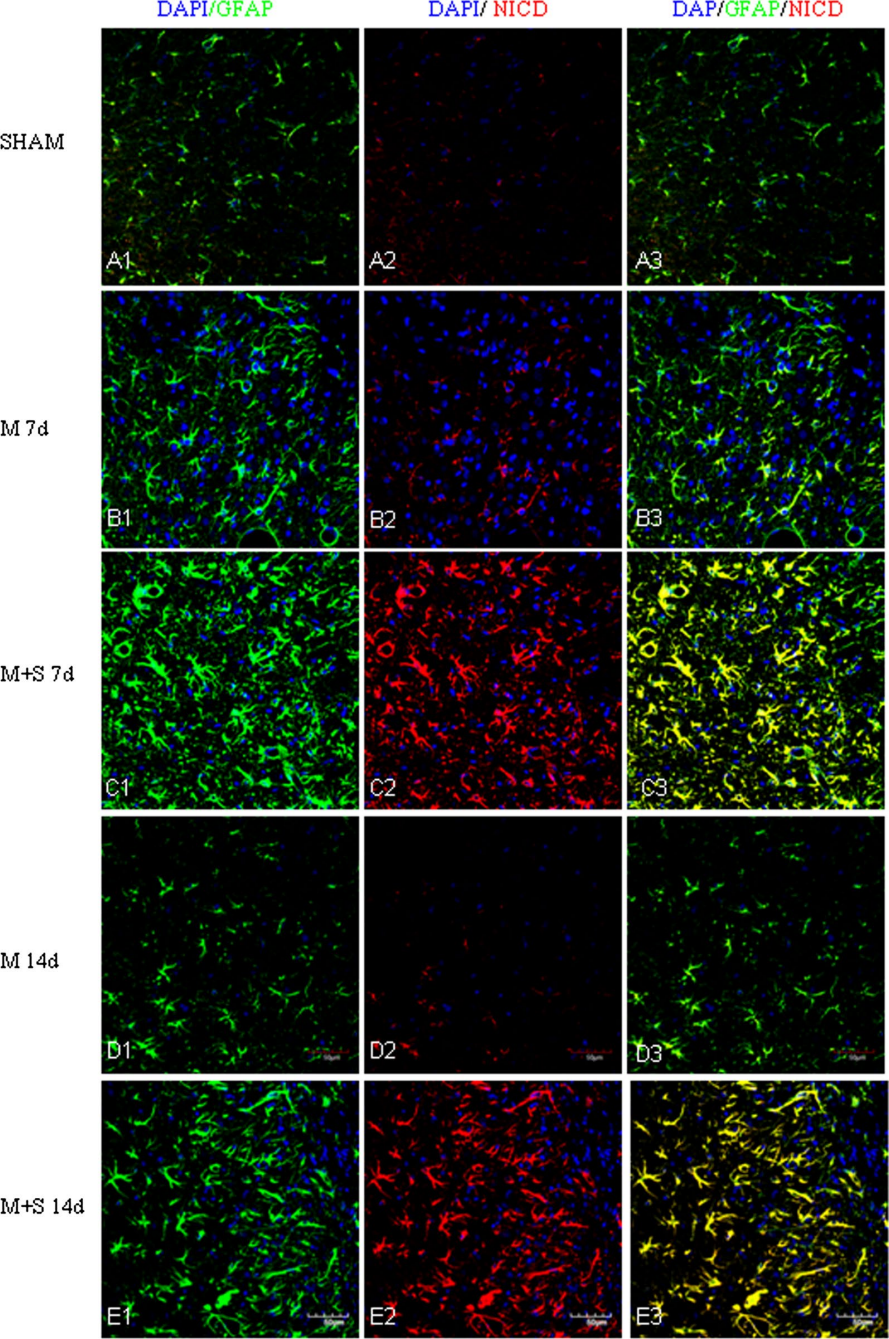
Scutellarin enhanced NICD expression in astrocytes after MCAO. NICD expression is absent in astrocytes in the sham (**A1**–**3**). Its expression (*red*) was moderately induced after MCAO in GFAP positive astrocytes (*green*) at 7 days (**B1**–**3**) but was diminished at 14 days (**D1**–**3**). However, NICD expression was markedly increased in hypertrophic astrocytes in scutellarin treated MCAO rats (**C1**–**3**, **E1**–**3**) in comparison to the matching control groups (**B1**–**3**, **D1**–**3**). *Scale bars*: 50 µm. DAPI-*blue*

**Fig. 8:**
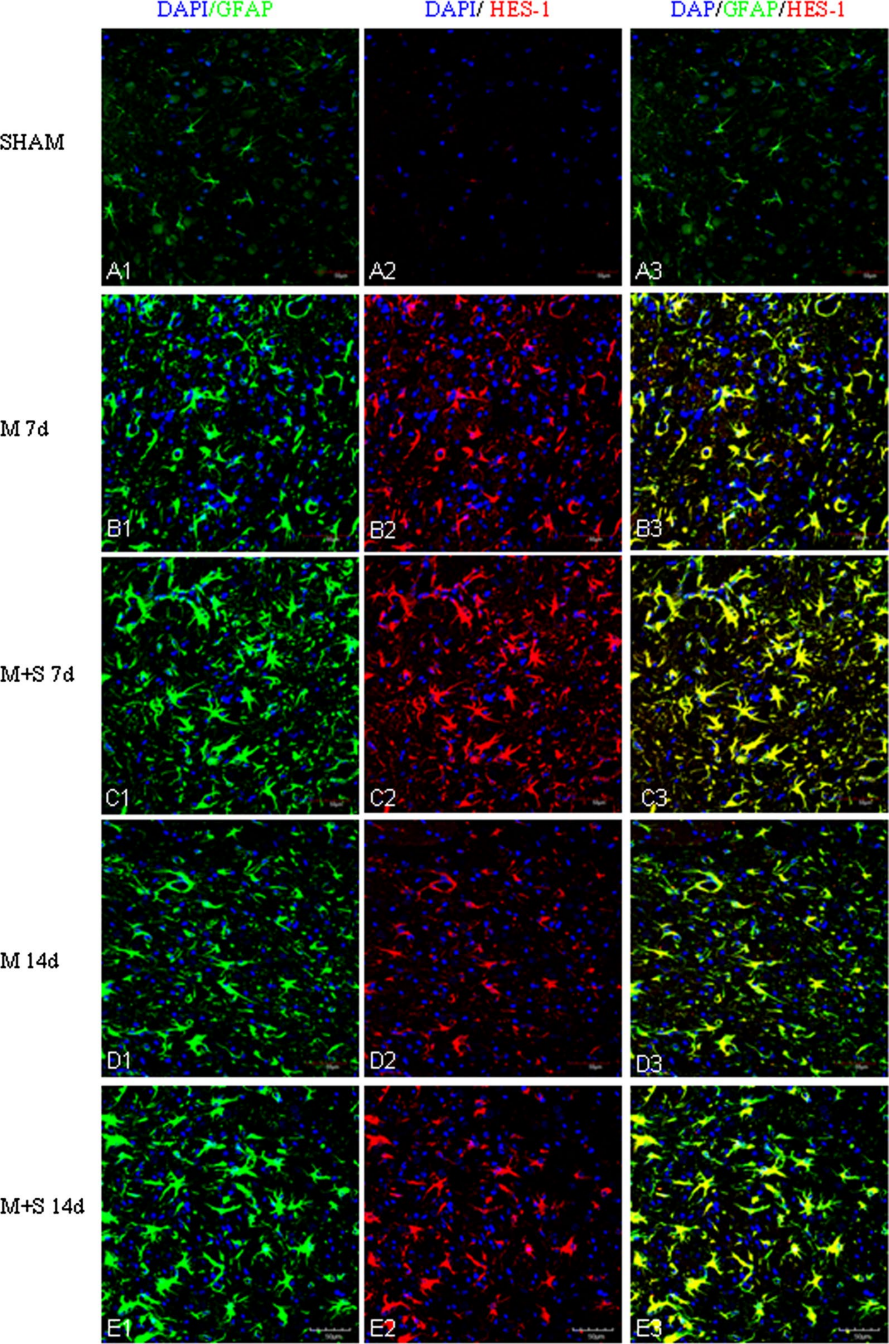
Scutellarin enhanced HES-1 expression in astrocytes after MCAO. HES-1 expression is undetected in astrocytes in the sham (**A1**–**3**). Its expression (*red*) was moderately induced after MCAO in GFAP positive astrocytes (*green*) at 7 days (**B1**–**3**) but was attenuated at 14 days (**D1**–**3**). However, HES-1 expression was markedly increased in hypertrophic astrocytes in scutellarin treated MCAO rats at 7 (**C1**–**3**) and 14 days (**E1**–**3**) in comparison to the matching control groups (**B1**–**3**, **D1**–**3**). *Scale bars*: 50 µm. DAPI-*blue*

**Fig. 9:**
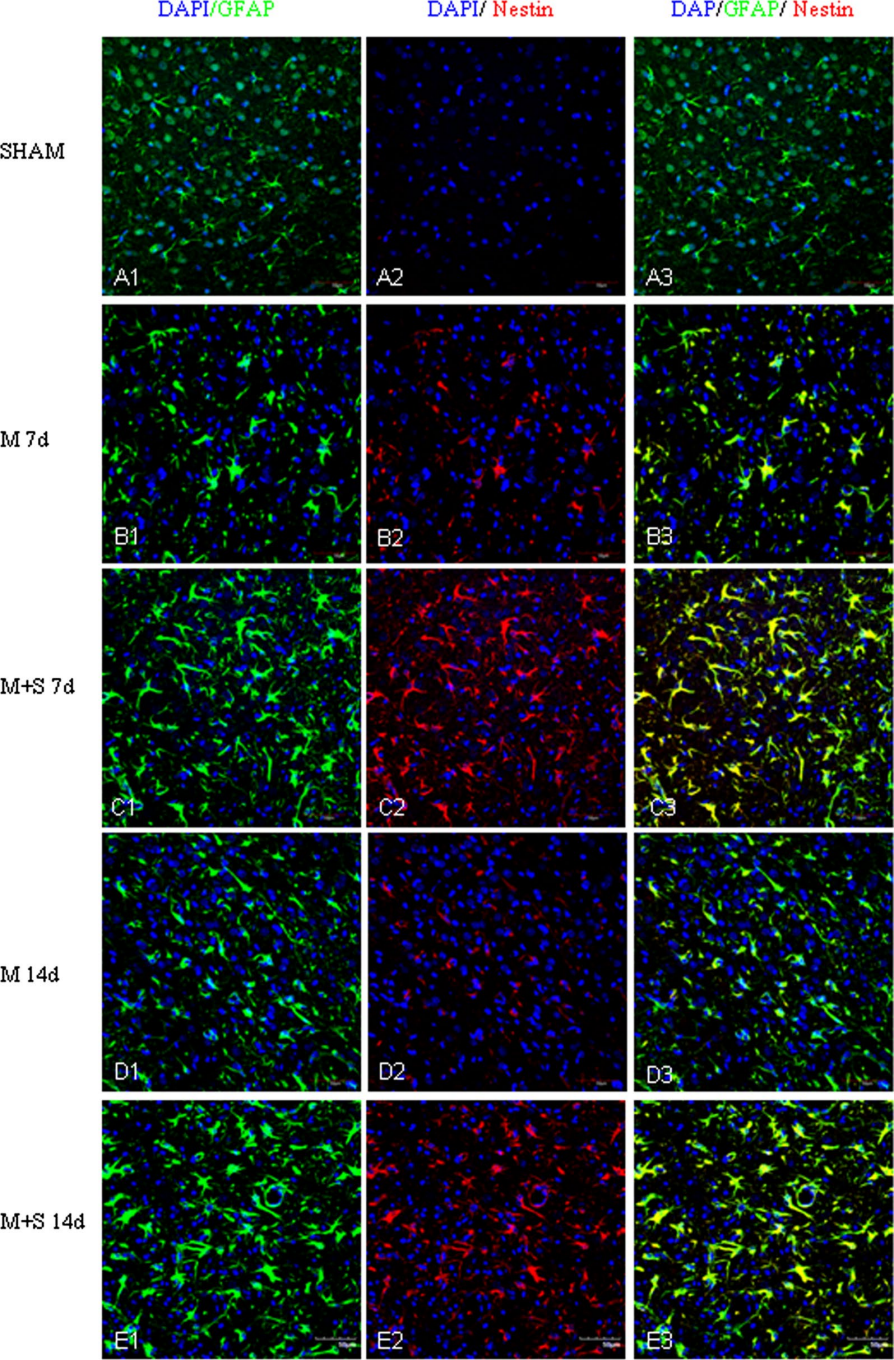
Scutellarin enhanced nestin expression in astrocytes after MCAO. Nestin expression is undetected in astrocytes in the sham (**A1**–**3**). It (*red*) was moderately induced in GFAP positive astrocytes (*green*) in MCAO rats at 7 (**B1**–**3**) but declined at 14 days (**D1**–**3**). However, nestin expression was markedly increased in hypertrophic astrocytes in scutellarin treated MCAO rats at 7 (**C1**–**3**) and 14 days (**E1**–**3**) in comparison to the matching control groups (**B1**–**3**, **D1**–**3**). *Scale bars*: 50 µm. DAPI-*blue*

**Fig. 10:**
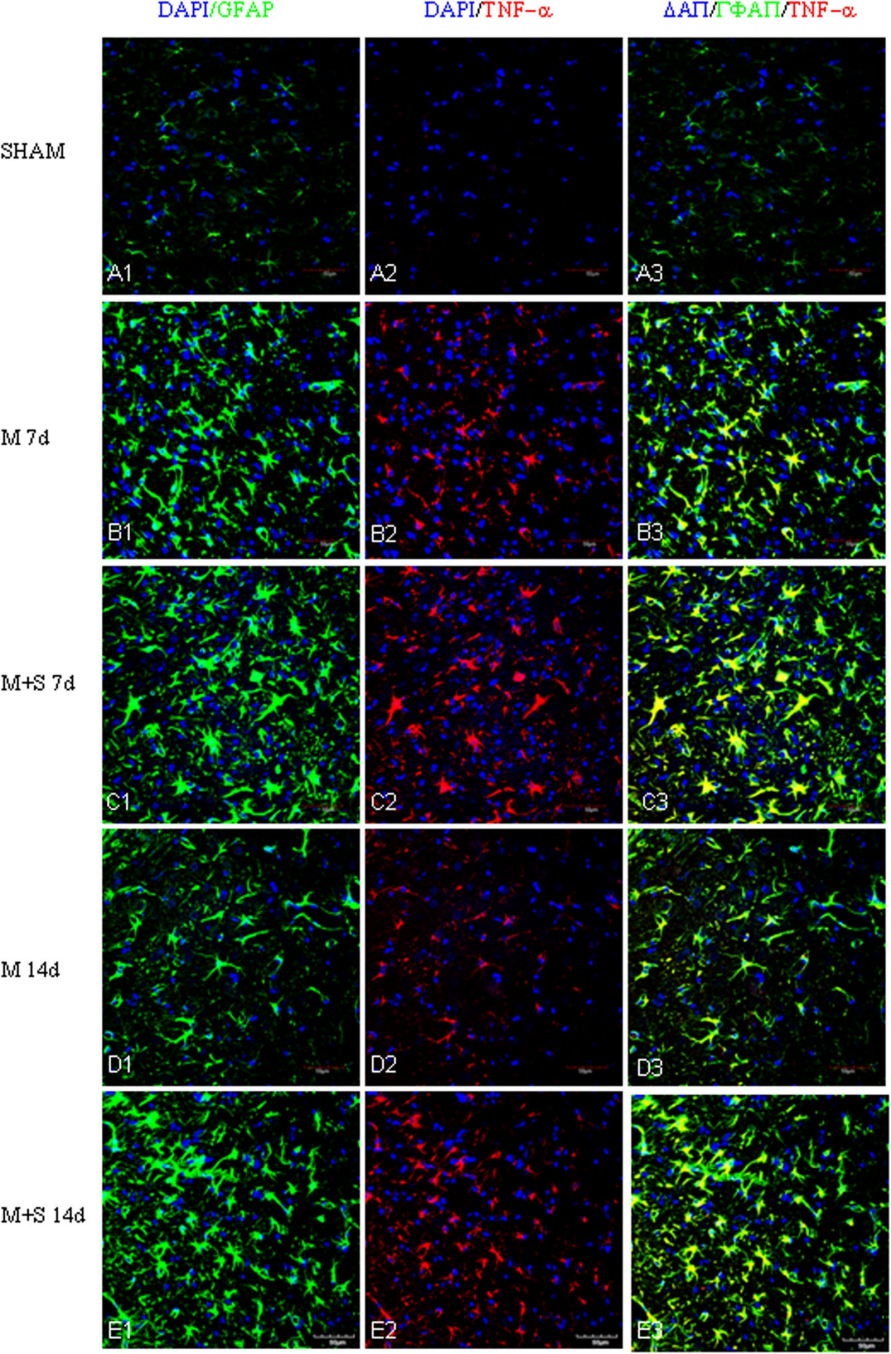
Scutellarin enhanced TNF-α expression in astrocytes after MCAO. TNF-α expression (*red*) was absent in astrocytes in the sham (**A1**–**3**). It was induced in GFAP positive astrocytes (*green*) at 3 and 7 days (**B1**–**3**) after MCAO, but the expression subsided at 14 days (**D1**–**3**). In MCAO rats treated with scutellarin (**C1**–**3**, **E1**–**3**). TNF-á expression in astrocytes was markedly enhanced being most pronounced at 7 days when compared with the matching MCAO control groups (**B1**–**3**, **D1**–**3**). *Scale bars*: 50 µm. DAPI-*blue*

By immunofluorescence microscopy, robust astrocyte reaction was observed in the penumbral region in ischemic cerebral cortex, notably at 7 and 14 days after MCAO. MCAO induced astrocyte reaction was evidenced by the enhanced GFAP labeling coupled with increased expression of Notch-1 (Fig. 6) and its members, NICD (Fig. 7) and HES-1 (Fig. 8). A striking feature after MCAO was the induced expression of nestin in GFAP positive astrocytes (Fig. 9). Expression of TNF-α (Fig. 10), IL-1β (Additional file 3) and iNOS (Additional file 4) was concomitantly augmented.

In MCAO rats given scutellarin injections, astrocytic reaction as manifested by the immunofluorescence of the above-mentioned markers was further enhanced. Astrocytes were grossly hypertrophic in comparison with the matching MCAO control rats without scutellarin or sham group. Scutellarin further elevated the MCAO-induced expression of Notch-1 (Fig. 6), NICD (Fig. 7), HES-1 (Fig. 8) and nestin (Fig. 9). In parallel to this, expression TNF-α (Fig. 10), IL-1β (Additional file 3) and iNOS (Additional file 4) immunofluorescence in the reactive astrocytes also became more pronounced. The enhanced increase for the above biomarkers by scutellarin was progressive when compared with counterpart cells in the MCAO and sham group being most pronounced at 7 days and was sustained till 14 days; thereafter, the expression appeared to diminish but remained to be more intense when compared with cells at the corresponding time-points at 21 days (Additional file 5).

Scutellarin not only accentuated the immunofluorescence of the various markers in reactive astrocytes, but also induced the changes of their phenotype. Thus, in MCAO rats given scutellarin treatment, the cells were evidently enlarged bearing dilated processes notably at 7 and 14 days (Figs. 6, 7, 8, 9, 10, Additional files 3, 4). GFAP labeled hypertrophic astrocytes often extended their long processes between activated microglia or encircled the outer walls of the blood vessels (Fig. 5).

## Discussion

### Direct effects of scutellarin on microglia and astrocytes

We reported recently that scutellarin could suppress NFkB and Notch signaling in activated microglia in their production of proinflammatory mediators in MCAO rats as well as in BV-2 cells [5, 6].This was sequel to our earlier demonstration that Notch-1 expression in activated microglia could transactivate NFkB that is linked to production of proinflammatory mediators [16–18]. In light of this, it was therefore concluded that scutellarin is a potential therapeutic agent that can attenuate microglia-mediated neuroinflammation in ischemic/hypoxic brain injury. It is well documented that associated with the microglia activation in ischemic injury, astrogliosis also featured prominently [19, 20]. Indeed, following diverse brain injuries, astrocytes are activated and described as reactive astrocytes [21–23]. Reactive astrocytes are known to undergo proliferation and are involved in different functions such as scar formation and tissue repair among others [19, 24]. Scutellarin could reduce the infarct area in rats with MCAO [5] indicating that it can promote tissue repair in ischemic injury and, hence, exert its effects on reactive astrocytes which play a pivotal role in this process. The purpose of this study was to ascertain if scutellarin would act on astrocytes, and if so, to determine its mode of action. For this, we first investigated the effect of scutellarin on TNC1 astrocytes in vitro. As opposed to BV-2 cells, direct application of scutellarin to TNC1 did not elicit an obvious increase in GFAP expression, a specific marker for astrocytes; moreover, neither TNF-α nor iNOS expression was noticeably altered. It stands to reason therefore that scutellarin does not act directly, if at all, on astrocytes. It was then surmised that scutellarin might act through intermediary cells. A putative candidate for this would be the microglia whose activation in the ischemic injury is acute in onset [25] and invariably precedes that of astrocytes; furthermore, activated microglia and reactive astrocytes at the injury site are in close proximity as is evident in the present MCAO model suggesting the possibility of their functional interaction.

### Microglia mediate astrocyte reaction

In consideration of a possible interaction between activated microglia and astrocytes, TNC1 astrocytes were first exposed to basic medium (BM), or basic medium + LPS (BM + L). Interestingly, GFAP, TNF-α and iNOS expression between the two TNC1 groups was moderate and comparable. This suggests that direct LPS treatment did not evoke a noticeable response in TNC1 astrocytes. Against this however was the report that LPS could activate the primary astrocytes bearing TLR4 [26]. The discrepancy in results may be attributed to different cell models used. Remarkably, when TNC1 astrocytes were exposed to conditioned medium derived from LPS-stimulated BV-2 cells (CM + L), a vigorous response was observed as manifested by the marked increased in GFAP, TNF-α and iNOS expression. As a corollary, it is suggested that the drastic TNC1 astrocyte reaction is mediated by activated microglia.

### Scutellarin amplifies microglia-mediated astrocyte reaction

The present results have shown that scutellarin is capable of amplifying the astrocyte reaction. This notion lends its support from the fact that GFAP, Notch-1, NICD, HES-1, nestin expression along with that of TNF-α, IL-1β and iNOS was noticeably increased in TNC1 astrocytes exposed to CM + SL, when compared with cells incubated in CM + L. By Western blot, the increase was in the range of 10–30 % of the expression levels across the various proteins. A similar increase in expression occurred in reactive astrocytes in MCAO rats given scutellarin treatment. In this, the reactive astrocytes exhibited more intense immunofluorescence of the above markers compared with MCAO rats not treated with the drug. Microglia-mediated astrocyte reaction is also evident by an apparent hypertrophy of astrocytes bearing long filamentous processes and expansion of cells as revealed by electron microscopy. The functional significance for the phenotypic change is uncertain but it may promote communication between reactive astrocytes via the intertwined filamentous in tissue repair. In MCAO, the long extending processes of reactive astrocytes were observed to intercalate between activated microglia. The close spatial relation between activated microglia and reactive astrocytes in the complex environment in ischemic tissue may facilitate a paracrine function. Indeed, it was reported that activated microglia through production of MCSF could stimulate astrocytes in their production of proinflammatory cytokines [15] suggesting the functional relation or communication between two glial cell types. Furthermore, it has been reported that the communication pathways among microglia, astrocytes and neurons are responsible for neuroprotection [27]. Such communication may be mediated by microglia ATP via P2Y1 receptor on astrocyte which resulted in production of IL-6 that provides neuroprotection. Crosstalk between macrophages and astrocytes affecting the proliferation, reactive phenotype and inflammatory response, suggesting a role during reactive gliosis following spinal cord injury has recently been reported [28].

### Scutellarin enhances GFAP, Notch-1 pathway and nestin expression in reactive astrocytes

Contrary to its suppression of microglia activation in MCAO model and BV-2 cells, we show here that scutellarin instead promotes the astrocyte reaction as shown by the enhanced expression of Notch-1, NICD and HES-1. Additionally, scutellarin expanded the expression of nestin in virtually every reactive astrocyte both in vitro and in vivo. Reactive astrogliosis is reported to be involved in tissue repair or scar formation. It is conceivable that increase in hypertrophic astrocytic processes with enhanced GFAP which constitutes the cytoskeleton or intermediate filaments would facilitate such a function. Furthermore, vascular profiles of varying sizes in the penumbral region appeared to be fully invested by astrocytic end-feet suggesting that the integrity of the blood–brain barrier which would have been compromised in ischemia is being restored.

Nestin is a marker of neural stem cells and neural progenitor cells, and its expression is observed in a subpopulation of reactive astrocytes [24, 29]. It is a class VI intermediate filament protein that was first described as a neural stem/progenitor cell marker [30]. Neuroepithelial stem cells can differentiate into neurons, oligodendrocytes, and astrocytes, and nestin has been shown to be down-regulated or to completely disappear during such differentiation. Here we showed that nestin which was hardly detected in TNC1 was induced in some cells in CM + L conditioned medium. In CM + SL treated cells, nestin expression was detected and markedly enhanced in all cells. Virtually all GFAP labeled reactive astrocytes in scutellarin injected MCAO rats emitted intense nestin immunofluorescence compared with the matching control at the corresponding time-points. The expression of nestin in reactive astrocytes suggests that some of them had “de-differentiated” and assumed a “stemness” property, but it remains to be determined if they might differentiate [31]. While reactive astrocytes appear to have greater plasticity than was previously realized [32], and may provide a source of multipotent cells or functional neurons for regenerative medicine, the signaling pathway(s) that govern the formation of injury-induced nestin positive reactive astrocytes having the potential of neural stem cells remains uncertain. Thus, the demonstration of Notch-1 signaling and its members both in vivo and in vitro in this study may provide a clue for the regulatory mechanism of nestin expression in reactive astrocytes. It is relevant therefore to note that proliferating reactive astrocytes are regulated by Notch-1 in the peri-infarct area after stroke [23]. It has been reported that treatment of MCAO mice with DAPT, an inhibitor of γ secretase, decreases the number of olig2+ and nestin+ reactive astrocytes [33] alluding to the possibility that Notch-1 signaling in reactive astrocytes regulates nestin expression. The colocalization and concurrent upregulation of Notch-1 signaling and nestin in reactive astrocytes in the present results suggest that both may operate in synergy; but in the lack of experimental evidence, this remains purely speculative. Other pathways or molecules such as miRNAs known to regulate astrogliosis [34–36], and that might be involved in microglia-mediated astrocyte reaction and modulated by scutellarin should be future scope of study.

### Scutellarin promotes TNF-α, IL-1β and iNOS in reactive astrocytes

While scutellarin decreases the expression of proinflammatory mediators including TNF-α, IL-1β, iNOS and NO production in activated microglia, it conversely upregulates their expression in reactive astrocytes. It is well documented that excess production of proinflammatory mediators from activated microglia is detrimental to neurons [37]; on the other hand, their increased production in reactive astrocytes by scutellarin remains speculative. There is increasing agreement that TNF-α in vitro and in vivo may protect neurons against excitotoxic, oxidative and ischemic injuries. It may be hypothesized that TNF-α is not potent to kill neurons by itself, but it may function synergistically with other cytokines and toxic agents such as NO, free radicals or glutamate [38]. Likewise, IL-1β can promote survival of retinal ganglion cells [39] and neurons [40]. Furthermore, it has been reported that nitric oxide could induce the expression of GFAP in reactive astrocytes which exhibit iNOS expression [41]. It has been reported that iNOS-derived NO can act as an endogenous antioxidant in traumatic brain injury suggesting the neuroprotective role of iNOS [42]. Taken together, it is suggested that the increased expression of TNF-α, IL-1β and iNOS by scutellarin might not necessary be neurotoxic, but can be neuroprotective. It may also be argued that the final result/amount of scutellarin-regulated pro-inflammatory mediators would determine whether they are neurotoxic or neuroprotective but this remains debatable. From a speculative point of view, TNF-α, IL-1β and iNOS derived from reactive astrocytes might be beneficial instead of cytotoxic as those from activated microglia.

## Conclusion

Scutellarin can regulate the expression of GFAP, Notch-1 signaling, and nestin along with that of proinflammatory mediators in reactive astrocytes in ischemic injury. In vitro results showed that scutellarin did not exert a direct effect on astrocytes; rather, it acted through the intermediary activated microglia. Scutellarin acts primarily to amplify the microglia-mediated astrocyte reaction thus highlighting a functional “cross-talk” between activated microglia and reactive astrocytes. On the other hand, the specific molecules such as cytokines involved in this are uncertain. Notwithstanding, it is concluded that scutellarin is a potent agent that can facilitate the communication between activated microglia and reactive astrocytes in ischemic injury, an inter-glial cell mechanism that may be crucial to issue repair.

## Methods

### Cell culture and in vitro study

DI TNC1, type 1 phenotype of *Rattus norvegicus* astrocyte purchased from American Type Culture Collection (ATCC, USA, CRL-2005™) was maintained in 75 cm^2^ culture flasks with completed medium composed of basic medium (Dulbecco’s Modified Eagle’s Medium, Sigma, St. Louis, MO, USA; Cat. No. 1152) and supplement with 10 % fetal bovine serum (FBS, HyClone, Logan, UT, USA). The cultures were incubated at 37 °C in a humidified incubator under 5 % CO_2_.

BV-2 cells (a widely used murine microglial cell line) were maintained in our laboratory under the same condition as for TNC1 for the production of conditioned medium.

### Cell viability assay of TNC1 astrocytes

Cell viability was assessed by CellTiter 96 W Aqueous One Solution Cell Proliferation Assay kit (Promega, Fitchburg, WI, USA; Cat. No. G3580). To determine the cytotoxic effect of scutellarin on TNC1, cells were plated into 96-well microplates (10^4^ cells/well) and cultured for 24 h. They were subjected to treatment of scutellarin (in the range of 0.2–2.0 mM) in each well containing 100 μl of culture medium for 1 h in triplicates. Briefly, 20 μl of MTS(3-(4,5-dimethylthiazol-2-yl)-5-(3-carboxymethoxyphenyl)-2-(4-sulfophenyl)-2H-tetrazolium, inner salt) reagent was added to each well (final concentration, 0.5 mg ml^−1^) and the plate was incubated for an additional 4 h. The optical density (OD) was then read at 490 nm using a microplate reader (GENIOS, Tecan, Switzerland). The assays were performed in triplicate.

### Preparation and use of BV-2 conditioned medium

At 24 h after BV-2 cell seeding, the medium was discarded. Three different kinds of conditioned medium were prepared: BV-2 conditioned medium as control (CM): incubation of BV-2 cells was in 10 mL basic DMEM for 3 h; BV-2 conditioned medium + lipopolycharride (LPS) (CM + L): BV-2 cells were incubated with 10 mL basic medium with 1 µg/ml of LPS for 3 h; BV-2 conditioned medium with scutellarin pretreatment + LPS (CM + SL): for this, BV-2 cells were first incubated with 10 ml basic medium containing 0.54 mM of scutellarin for 1 h. This dosage was used based on the cell viability assay and the dose-dependency assay by us previously [5, 6]; the medium was then discarded and the cells were washed with PBS twice. Following this, 10 ml basic medium containing 1 µg/ml of LPS was added for another 3 h. All 3 conditioned media were collected and filtered through 0.22 μm syringe filters; they were then ready to use for treatment of TNC1 astrocytes.

### TNC1 astrocytes and treatment with BV-2 conditioned medium

TNC1 astrocytes were divided into control (CM), LPS-induced (CM + L) and scutellarin + LPS (CM + SL) groups based on treatment with the respective conditioned medium. TNC1 cells were seeded at a density of 3.0 × 10^5^ in 6-well plates and 5.0 × 10^4^ in 24-well plates, incubated with a complete medium. After 24 h, the medium was discarded and the cells were incubated with 2 ml (6-well plates) or 1 ml (24-well plates) of conditioned medium for 24 h. The cells were then harvested for protein extraction and RNA isolation (6-well plates) or immunofluorescence microscopy (24-well plates).

### RNA isolation and real-time RT-PCR for validation of TNCI reaction

Total RNA from TNC1 astrocytes was extracted using miRNeasy Mini Kit (Qiagen, Germany, Cat. No. 217004) according to the manufacturer’s instructions. After this, 1 μg of RNA from each sample was added to a total volume of 20 μl reaction mixture containing 0.5 μg of oligo (dT) primer (Promega, Madison, WI, USA; Cat. No. A3500), and 15U of AMV Reverse Transcriptase (Promega, Madison, WI, USA; Cat. No. M5314). The reaction was initiated by incubating the reaction mixture for 15 min at 42 °C, then heating at 95 °C for 5 min, and stopped by incubating at 0–5 °C for 5 min. Aliquot (3 μl) of each of the diluted reverse transcription products (diluted by 1:5) was added to the 10 μl reaction mixture containing 5 μl of GoTaq^@^qPCR Master Mix (Promega, Madison, WI, USA; Cat. No. A6001/2), 0.5 μM of each primer corresponding to TNF-α, iNOS and β-actin, to amplify the genes in ABi 7900HT Fast PCR system (Applied Biosystems, USA). The primer sequences of TNF-α are forward: 5′-TATGGCTCAGGGTCCAACTC-3′ and reverse: 5′-CTCCCTTTGCAGAACTCAGG -3′, iNOS are forward: 5′- CTCACTGGGACAGCA CAGAA -3′ and reverse: 5′- GCTTGTCTCTGGGTCCTCTG-3′, β-actin are forward: 5′-AGCCATGTACGTAGCCATCC-3′ and reverse: 5′-GCTGTGGTGGTGAAGCTGTA-3′. After pre-incubation at 95 °C for 15 min, the polymerase chain reaction (PCR) was performed as follows: 40 cycles of denaturation at 94 °C for 15 s, annealing at 57 °C for 25 s, and elongation at 72 °C for 15 s.

### Western blotting analysis for DI TNC1 astrocytes

After incubating with different media for 24 h, TNC1 astrocytes of different groups (CM, CM + L and CM + SL) were treated with lysis buffer after rinsing twice with 1xPBS. Cell lysate was mechanically scrapped off and centrifuged at 14,000 rpm for 15 min and the supernatant was collected. Protein concentrations of TNC1 astrocytes were determined with a colorimetric protein assay by protein assay kit (Bio-Rad, Hercules, CA, USA; Cat. No. 500-0002). Protein samples were then heated with 6x protein loading buffer at 95 °C for 5 min to denature the protein. Thirty μg of each protein sample was separated on sodium dodecyl sulfate–polyacrylamide gel electrophoresis (SDS-PAGE) with 10 % gels, in a Mini-Protein II apparatus (Bio-Rad, CA, USA). Protein bands were electroblotted onto polyvinylindene difluoride (PVDF) membrane and blocked with non-fat dried milk for 1 h. The membranes were incubated with TNF-α (rabbit polyclonal IgG 1:1500) (Sigma-Aldrich; Cat. No. T8300), IL-1β (rabbit polyclonal IgG 1:500) (Santa Cruz Biotechnology, Cat. No. sc-7884), iNOS (rabbit polyclonal IgG 1:1000) (Thermo scientific, Cat. No. PA3-030A), Notch-1 (rabbit polyclonal IgG 1:500) (Santa Cruz Biotechnology, Cat. No. sc-6014-R), NICD (Rabbit polyclonal IgG 1:1000) (Merck KGaA, Cat. No. 07-1232), HES-1 (rabbit polyclonal IgG 1:300) (Santa Cruz Biotechnology, Cat. No. sc-25392) and β-actin (mouse monoclonal IgG 1:10000) (Sigma; Cat. No. A5441). Primary antibodies (Table 2) diluted in Tris-Buffered Saline-0.1 %Tween (TBST) overnight at 4 °C before the membranes were incubated with the secondary antibodies, either with horseradish peroxidase conjugated anti-rabbit IgG (ThermoScientific; Cat. No. 31460), or anti-mouse IgG (ThermoScientific; Cat. No. 31430). Protein was detected by a chemiluminescence kit (GE Healthcare UK Limited, Bucks, UK) following the manufacturer’s instructions and developed on film. The band intensity was quantified in Image J software (National Institutes of Health, NIH, USA). All experiments were repeated at least in triplicate.

**Table 2:** Antibodies used for western blotting and immunostaining

Antibody	Host	Source	Catalog number	RRIDs
TNF-α	Rabbit polyclonal	Sigma-Aldrich, USA	T8300	AB_477588
IL-1β	Rabbit polyclonal	Santa Cruz Biotechnology, CA, USA	sc-7884	AB_2124476
iNOS	Rabbit polyclonal	Thermo scientific, CA, USA	PA3-030A	AB_2152737
GFAP	Mouse monoclonal	Merck KGaA, Darmstadt, Germany	MAB360	AB_11212597
Nestin	Rabbit polyclonal	Sigma-Aldrich, USA	SAB4200347	
Notch-1	Rabbit polyclonal	Santa Cruz Biotechnology, CA, USA	sc-6014-R	AB_650335
NICD	Rabbit polyclonal	Merck KGaA, Darmstadt, Germany	07-1232	AB_1977387
Hes-1	Rabbit polyclonal	Santa Cruz Biotechnology, CA, USA	sc-25392	AB_647996
β-actin	Mouse Monoclonal	Sigma-Aldrich, MO, USA	A-5441	AB_476744

### Immunofluorescence labeling of TNC1 in different treatments

About 5 × 10^4^ TNC1 astrocytes were seeded on poly-l-lysine (Sigma, Cat. NO. P4707) pre-coated coverslips in a 24-well plate and left for 24 h to attach to the coverslip surface before various treatments. TNC1cells were divided into 3 groups as described above: CM, CM + L and CM + SL. After treatment with the conditioned medium from the respective group for 24 h, TNC1 astrocytes were fixed in 4 % paraformaldehyde for 15 min at room temperature followed by blocking with 5 % goat serum for 1 h after washing with 1xPBS for 3 times at 5 min each. Subsequently the cells were incubated with the respective primary antibodies (Table 2) diluted in 1xPBS (1:100–500) at 4 °C overnight. Antibodies were detected with the relevant FITC/Cy3-conjugated secondary antibodies (1:200, diluted by 1× PBS) for 1 h at room temperature. The coverslips were then mounted in DAPI containing the mounting medium (Sigma, Cat. No. F6057) after rinsing in 1× PBS. Images were captured using a confocal microscope (FLUOVIEW FV1000; Olympus, Japan).

### Electron microscopy

For scanning electron microscopy (SEM), TNC1 astrocytes from CM, CM + L and CM + SL were seeded on cover-slips in a 24-well plate at 2 × 10^4^/cm^2^. They were fixed in 2 % paraformaldehyde and 3 % glutaraldehyde in 0.1 M phosphate buffer at 4 °C for 1 h before postfixation with 1 % osmium tetroxide in PB, pH 7.4 for 30 min. After rinsing in 0.1 M PB, the cell samples were dehydrated through an ascending series of ethanol before being transferred to a Bal-Tec CPD-030 critical point dryer (Bal-Tec AG, Balzers, Liechtenstein), using liquefied carbon dioxide as the transition fluid. The cover slips were then mounted on SEM stub. All cells were sputter-coated with 20 nm gold in a sputter coater (Balzers SCD 004) before examination in a scanning electron microscope (FEI 650 SEM).

For transmission electron microscopy (TEM), TNC1 cells from the respective groups were fixed in 2 % paraformaldehyde and 3 % glutaraldehyde in PB at 4 °C for 1 h before post-fixation with 1 % osmium tetroxide, pH 7.4 for 1 h. After dehydration, the cells were embedded in resin which was allowed to polymerize at 60 °C for 24 h. Ultrathin sections were mounted on formvar-coated copper grids and double stained with uranyl acetate and lead citrate. The grids were viewed in a JEOL 1010 transmission electron microscope.

## Animal study

### Ethics statement on use of animals

This part of the study was carried out within an appropriate ethical framework. While handling and use of rats, ethical guidelines as stated in the National Institutes of Health Guide for the Care and Use of Laboratory Animals were strictly adhered to. All experimental protocols and use of animals were approved by Kunming Medical University (KMU), and all efforts were made to minimize the number of rats used and their suffering.

### Animals, surgical procedure, injection of scutellarin and animal groups

A total of 65 adult male Sprague–Dawley rats weighing 250–280 g were obtained from the Experimental Animal Center of Kunming Medical University. All surgical procedures were carried out in the Department of Anatomy and Histology/Embryology, KMU. The surgical procedures for middle cerebral artery occlusion (MCAO) followed that described previously by us [43, 44]. Briefly, the animals were anaesthetized with an intraperitoneal injection of sodium pentobarbital (50 mg/kg). After this, a circular aperture 3 mm in diameter was burred in the right parietal bone with a dental drill, and the main trunk of the middle cerebral artery (MCA) exposed and cauterized. In the sham-operated rats, the same surgical procedure was followed but the MCA was not cauterized. All efforts were made to minimize the number of rats used and their suffering. The rats were randomly divided into sham-operated + saline (sham), MCAO + saline (MCAO), and MCAO + scutellarin (100 mg/kg) (MCAO + scutellarin) groups (Table 3). The rats in scutellarin treated groups were given an intraperitoneal injection of scutellarin (100 mg/kg dissolved in saline Cat. No.131021,Shanghai, China) at 2 h before and at 12, 24, 36, 48, and 60 h after MCAO; rats were euthanized at 1, 3, 7, 14 and 21 days after MCAO.

**Table 3:** Number of rats used in various treatments

SHAM (Sham-operated + saline)	MCAO (MCAO + saline)	S + MCAO (Scutellarin + MCAO)
n = 15	n = 25	n = 25

### Double immunofluorescence labeling of MCAO brain sections

The rats from the MCAO groups at different time-points after scutellarin injections (n = 5 for each time-point) along with sham operated (n = 3 at each time point) and MCAO + saline injection (n = 5 for each time-point) serving as the controls were used for double immunofluorescence labeling. Following deep anesthesia with 6 % sodium pentobarbital, the rats were euthanized by perfusion with 2 % paraformaldehyde in 0.1 M phosphate buffer. The brain was removed and paraffin embedded. Coronal sections of 7 μm thickness were cut on a microtome (Model: 2165; Leica, Bensheim, Germany). For blocking of nonspecific binding proteins, tissue sections were incubated in 5 % normal goat serum diluted in phosphate-buffered saline (PBS) for 1 h at room temperature (22–24 °C). The sections were then incubated in a humidified chamber with the following primary antibodies against proinflammatory mediators: TNF-α (rabbit polyclonal IgG1:100) (Chemicon International, Temecula, CA, USA; Cat. No. AB1837P), IL-1β (rabbit polyclonal IgG 1:100) (Chemicon International; Cat. No. AB1832P) and iNOS (mouse monoclonal IgG 1:100) (BD Pharmingen, San Jose, CA USA; Cat. No. 610432), diluted with PBS overnight at 4 °C. Some sections were incubated in primary antibodies directed against the Notch signaling and its members including Notch-1 (rabbit polyclonal IgG 1:100) (Santa Cruz Biotechnology, Cat. No. sc-6014-R); intracellular Notch receptor domain, NICD (Rabbit polyclonal IgG 1:200) (Merck KGaA, Cat. No. 07-1232); transcription factor hairy and enhancer of split-1, HES-1 (rabbit polyclonal IgG 1:100) (Santa Cruz Biotechnology, Cat. No.sc-25392) diluted in PBS overnight at 4 °C. Following washing in PBS, sections were incubated, with the respective fluorescent secondary antibodies: Cy3-conjugatedsecondary antibody or FITC-conjugated lectin (*Lycopersicon esculentum*), which labels both microglia and blood vessel endothelial cells for 1 h at room temperature. Brain sections from different groups were also incubated with the primary antibodies against glial fibrillary acidic protein (GFAP), a specific marker for astrocytes (Merck KGaA, Darmstadt, Germany. Cat. No. MAB360), and nestin, a marker for neural stem cells (Sigma-Aldrich, MO, USA, Cat. No. SAB4200347). After three rinses with PBS, the sections were mounted with a fluorescent mounting medium containing 4′,6-diamidino-2-phenylindole (DAPI) (Sigma, MO, USA; Cat. No. F6057). Colocalization was observed by confocal microscopy (Fluoview1000, Olympus Company Pte. Ltd., Tokyo, Japan). The details of the antibodies used are given in Table 2. The region for all staining tests in MCAO rats was at the border areas of the infarct epicenter of ipsilateral ischemic cerebral cortex.

### Statistical analysis

Statistical analysis was performed by SPSS 16.0 statistical software. The data were expressed as mean ± SD. After homogeneity test of variances, one-way Analysis of Variance (ANOVA) followed by multiple comparison of Dunnet’s test was used to determine the statistical significance of different groups. All experiments were conducted in triplicate from different cell or tissue samples. The difference was considered statistically significant when P <0.05.

## Additional files

10.1186/s12868-015-0219-6 (A). Scutellarin at 0.54 mM did not elicit a noticeable reaction of GFAP/iNOS in TNC1. (B). iNOS mRNA expression in TNC1 astrocytes remained relatively unchanged at all time-points following treatment with BM, BM + L and CM; however, when incubated with CM + L for various time points, TNC1 showed a remarkable increase in iNOS peaking at 24 h. (C). Confocal images showing iNOS (C1-3) expression in TNC1 astrocytes incubated with different medium for 24 h. Compared with cells incubated in BM (C1) and BM + L (C2), TNC1 astrocytes incubated with CM + L (C3) were hypertrophic and showed a marked increase in iNOS immunofluorescence. Scale bars: 20 μm. DAPI—blue.
10.1186/s12868-015-0219-6 Scutellarin enhanced IL-1β (A1, A2, A3) and iNOS (B1, B2, B3) expression in TNC1 via BV-2-conditioned medium. In TNC1 astrocytes treated with CM, moderate expression of IL-1β and iNOS was detected (A1, B1). The expression was noticeably increased in CM + L (A2, B2) and further enhanced upon incubation with CM + SL for 24 h (A3, B3) with long cytoplasmic processes with expansions projected by TNC1 astrocytes (A3, B3). Scale bars: 20 μm.
10.1186/s12868-015-0219-6 Scutellarin enhanced IL-1β expression in astrocytes after MCAO. IL-1β expression was undetected in astrocytes in the sham (A1-3). Its expression (red) was noticeably induced in GFAP positive astrocytes (green) at 3 and 7 days (B1-3) after MCAO. However, the increase subsided at 14 days (D1-3). In MCAO rats treated with scutellarin (C1-3, E1-3), IL-1β expression in astrocytes was further enhanced, notably at 14 days, when compared with the respective MCAO control groups (B1-3, D1-3). Scale bars: 50 µm. DAPI-blue.
10.1186/s12868-015-0219-6 Scutellarin enhanced iNOS expression in astrocytes after MCAO. iNOS expression was undetected in astrocytes in the sham (A1-3). Its expression (red) was moderately induced in GFAP positive astrocytes (green) at 7 (B1-3) and 14 days (D1-3) after MCAO. In MCAO rats treated with scutellarin (C1-3, E1-3), iNOS expression in hypertrophic astrocytes was further enhanced, when compared with the respective MCAO control groups (B1-3, D1-3). Scale bars: 50 µm. DAPI-blue.
10.1186/s12868-015-0219-6 Showing Notch-1, NICD, HES-1, Nestin, TNF-α, IL-1β and iNOS expression (red) in GFAP (green) reactive astrocytes at 21 d after MCAO (M) and after scutellarin treatment (M + S). Note that the expression is diminished in MCAO but remained more intense with scutellarin treatment. Scale bars: 50 µm. DAPI-blue.
