# Supplementary figures and images for: Scutellarin regulates microglia-mediated TNC1 astrocytic reaction and astrogliosis in cerebral ischemia in the adult rats
Source: BMC Neurosci. 2015 Nov 25;16:84. doi: 10.1186/s12868-015-0219-6 (PMC4660684; doi:10.1186/s12868-015-0219-6)

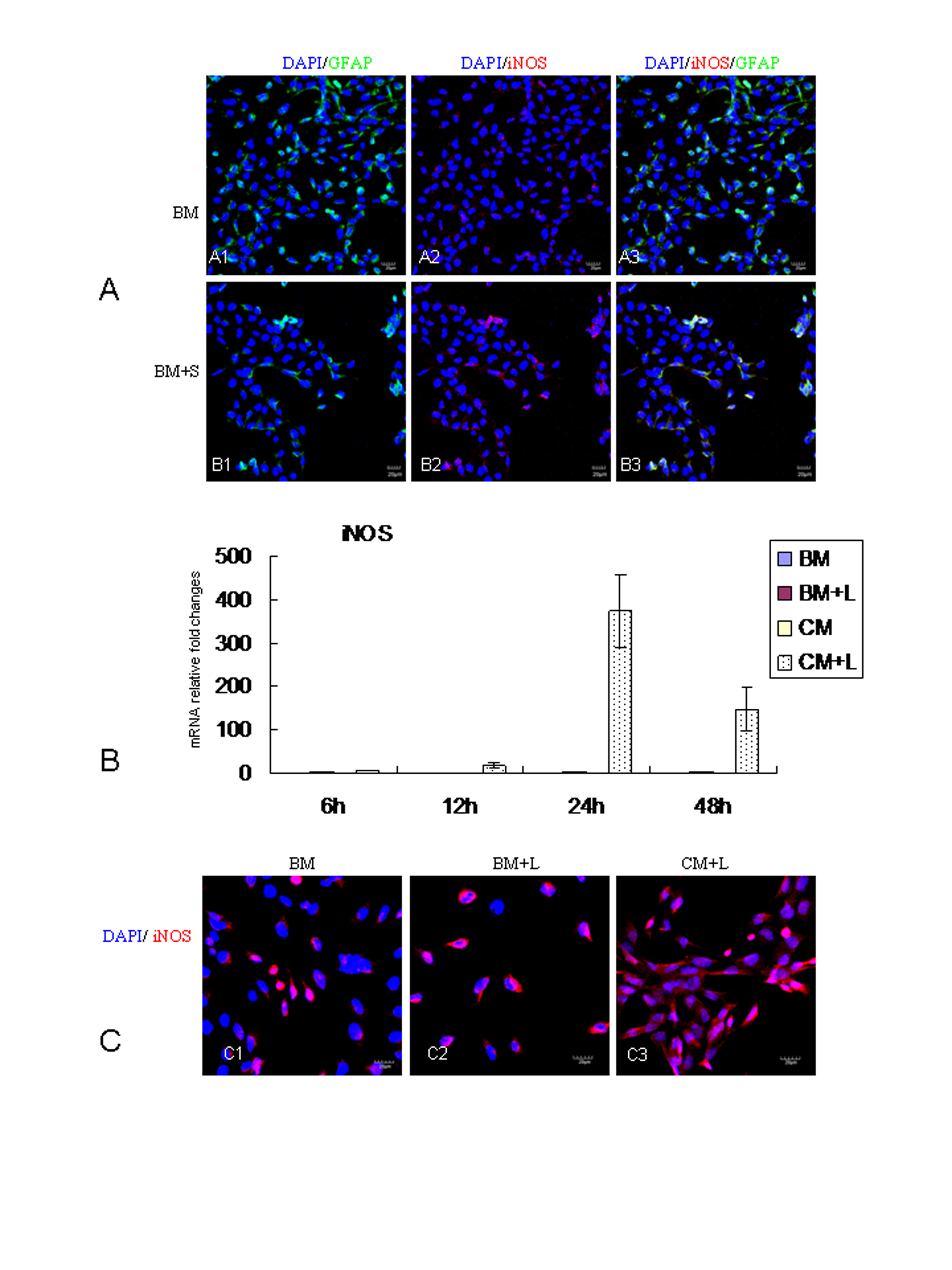

Supplement: Supplementary file 1 — 10.1186/s12868-015-0219-6 (A). Scutellarin at 0.54 mM did not elicit a noticeable reaction of GFAP/iNOS in TNC1. (B). iNOS mRNA expression in TNC1 astrocytes remained relatively unchanged at all time-points following treatment with BM, BM + L and CM; however, when incubated with CM + L for various time points, TNC1 showed a remarkable increase in iNOS peaking at 24 h. (C). Confocal images showing iNOS (C1-3) expression in TNC1 astrocytes incubated with different medium for 24 h. Compared with cells incubated in BM (C1) and BM + L (C2), TNC1 astrocytes incubated with CM + L (C3) were hypertrophic and showed a marked increase in iNOS immunofluorescence. Scale bars: 20 μm. DAPI—blue. [file 12868_2015_219_MOESM1_ESM.tif]

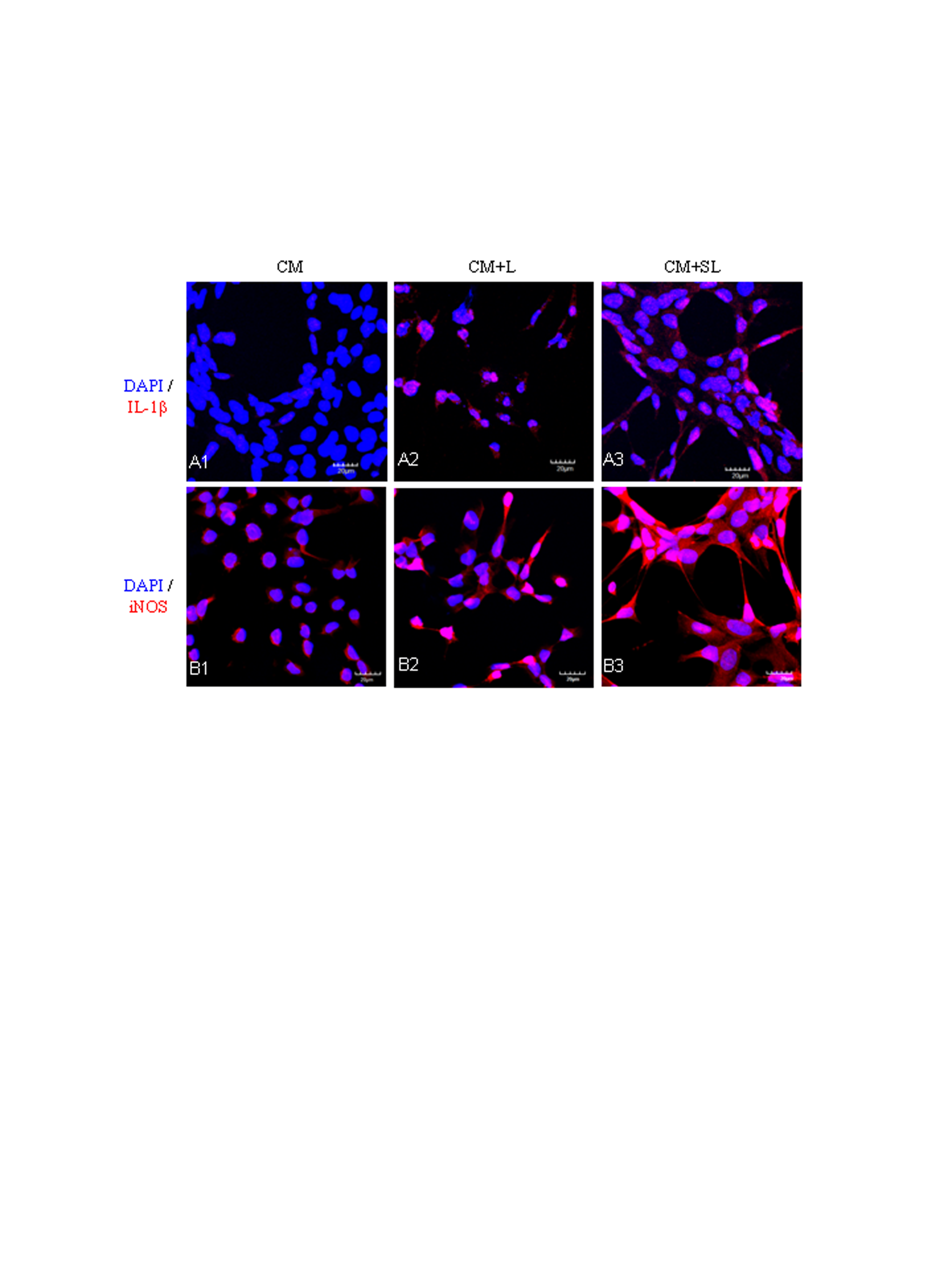

Supplement: Supplementary file 2 — 10.1186/s12868-015-0219-6 Scutellarin enhanced IL-1β (A1, A2, A3) and iNOS (B1, B2, B3) expression in TNC1 via BV-2-conditioned medium. In TNC1 astrocytes treated with CM, moderate expression of IL-1β and iNOS was detected (A1, B1). The expression was noticeably increased in CM + L (A2, B2) and further enhanced upon incubation with CM + SL for 24 h (A3, B3) with long cytoplasmic processes with expansions projected by TNC1 astrocytes (A3, B3). Scale bars: 20 μm. [file 12868_2015_219_MOESM2_ESM.tif]

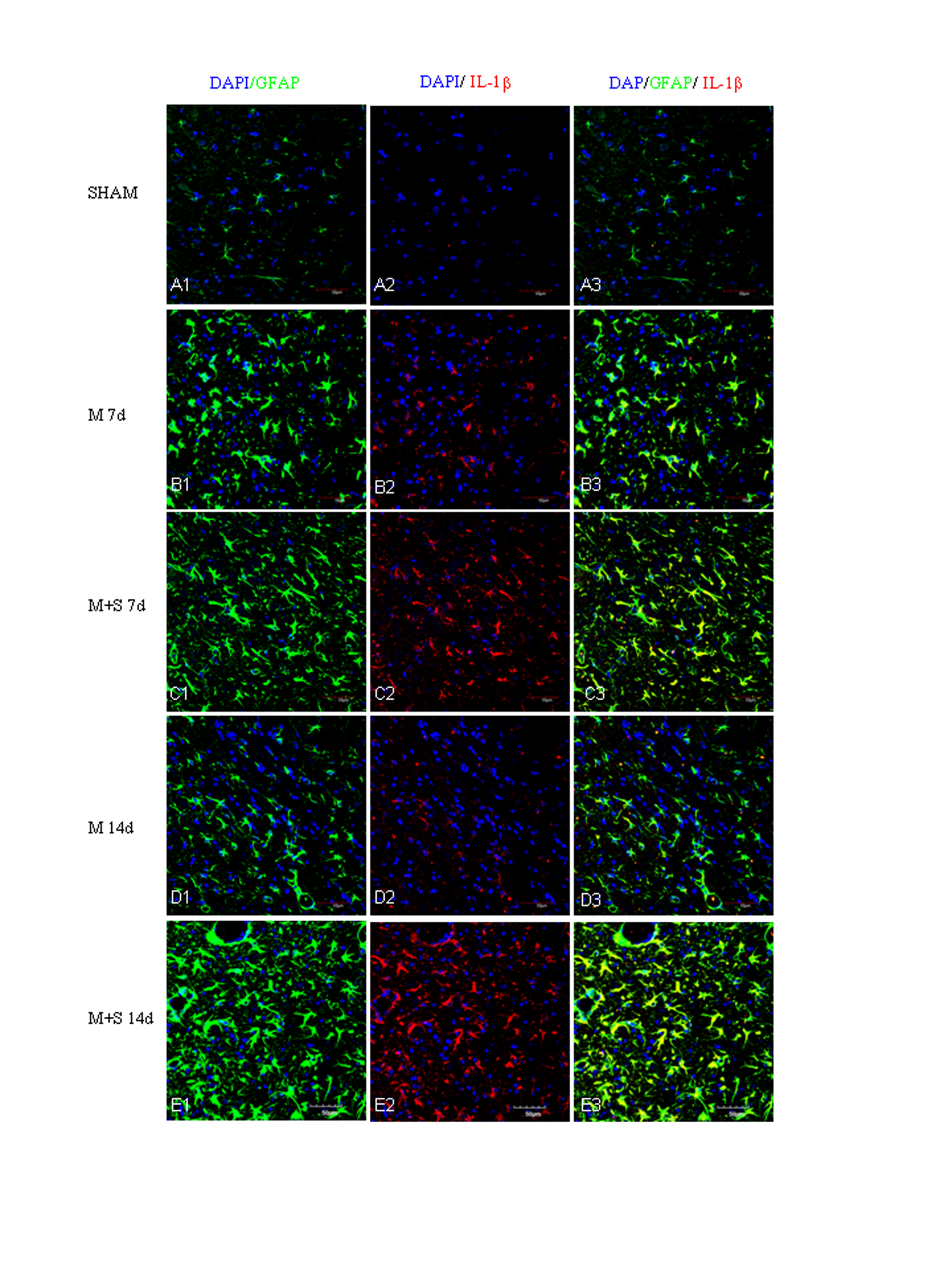

Supplement: Supplementary file 3 — 10.1186/s12868-015-0219-6 Scutellarin enhanced IL-1β expression in astrocytes after MCAO. IL-1β expression was undetected in astrocytes in the sham (A1-3). Its expression (red) was noticeably induced in GFAP positive astrocytes (green) at 3 and 7 days (B1-3) after MCAO. However, the increase subsided at 14 days (D1-3). In MCAO rats treated with scutellarin (C1-3, E1-3), IL-1β expression in astrocytes was further enhanced, notably at 14 days, when compared with the respective MCAO control groups (B1-3, D1-3). Scale bars: 50 µm. DAPI-blue. [file 12868_2015_219_MOESM3_ESM.tif]

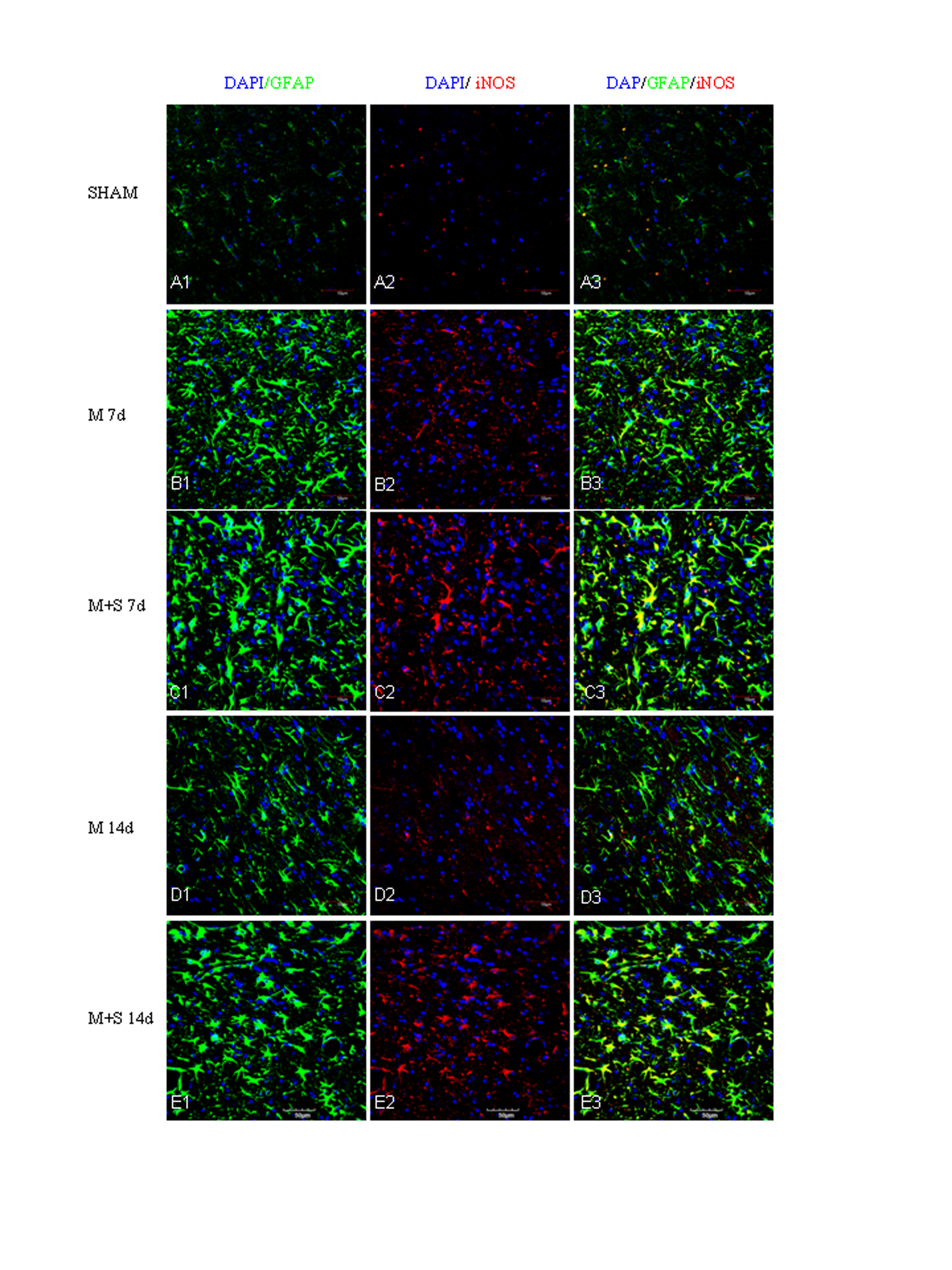

Supplement: Supplementary file 4 — 10.1186/s12868-015-0219-6 Scutellarin enhanced iNOS expression in astrocytes after MCAO. iNOS expression was undetected in astrocytes in the sham (A1-3). Its expression (red) was moderately induced in GFAP positive astrocytes (green) at 7 (B1-3) and 14 days (D1-3) after MCAO. In MCAO rats treated with scutellarin (C1-3, E1-3), iNOS expression in hypertrophic astrocytes was further enhanced, when compared with the respective MCAO control groups (B1-3, D1-3). Scale bars: 50 µm. DAPI-blue. [file 12868_2015_219_MOESM4_ESM.tif]

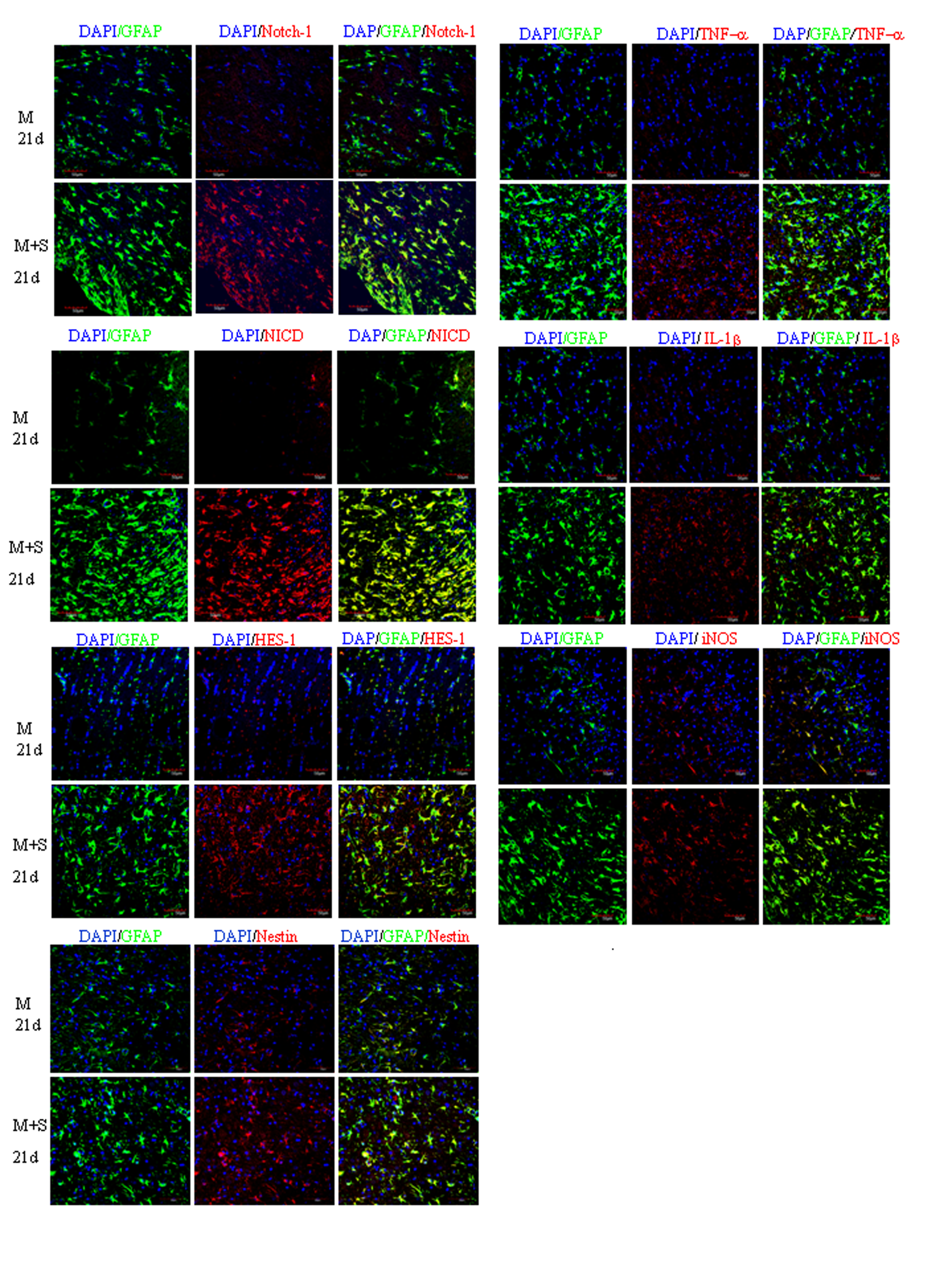

Supplement: Supplementary file 5 — 10.1186/s12868-015-0219-6 Showing Notch-1, NICD, HES-1, Nestin, TNF-α, IL-1β and iNOS expression (red) in GFAP (green) reactive astrocytes at 21 d after MCAO (M) and after scutellarin treatment (M + S). Note that the expression is diminished in MCAO but remained more intense with scutellarin treatment. Scale bars: 50 µm. DAPI-blue. [file 12868_2015_219_MOESM5_ESM.tif]
